# Hybrid Application of RSM and ANN to Optimize Natural Deep Eutectic Solvent-Based Distillation of *Ocimum basilicum* Essential Oil and Its Biological Activities

**DOI:** 10.3390/ijms27177692

**Published:** 2026-08-27

**Authors:** Iqra Khan, Sumia Akram, Zainab Liaqat, Faizan Waseem Butt, Haroon Iftikhar, Noor Ul Ain Khalid, Rizwan Ashraf, Imad A. Abu-Yousef, Amin F. Majdalawieh, Raed A. Al-Qawasmeh, Mohamed El-Naggar, Muhammad Mushtaq

**Affiliations:** 1 Division of Science and Technology, University of Education, Lahore-54000, Pakistansumia.akram@ua.edu.pk (S.A.); 2 Department of Chemistry, Government College University, Lahore-54770, Pakistan; zainabliaqat1@gmail.com (Z.L.); ravianfaizan97@gmail.com (F.W.B.); chemistharoon@yahoo.com (H.I.); chemistnoor@yahoo.com (N.U.A.K.); 3 Department of Biology, Chemistry and Environmental Sciences, American University of Sharjah, Sharjah P.O. Box 26666, United Arab Emirates; rashraf@aus.edu (R.A.); amajdalawieh@aus.edu (A.F.M.); 4 Department of Chemistry, University of Agriculture, Faisalabad 38040, Pakistan; 5 Department of Chemistry, University of Sharjah, Sharjah P.O. Box 27272, United Arab Emirates; ralqawasmeh@sharjah.ac.ae (R.A.A.-Q.); melnagrr@sharjah.ac.ae (M.E.-N.); 6 Advanced Biotechnology Center, Research Institute for Sciences and Engineering, University of Sharjah, Sharjah P.O. Box 27272, United Arab Emirates; 7 Department of Chemistry, The University of Jordan, Amman 11942, Jordan

**Keywords:** natural deep eutectic solvent, essential oil, optimization of extraction, artificial neural network, biological activities

## Abstract

The extraction of essential oil from agricultural resources continues to be a challenge in the context of purity, the integrity of delicate compounds, and low yield. In the current study, a new distillation protocol, involving the use of Glucose–Citric Acid-based Natural Deep Eutectic Solvent (NaDES), has been developed and optimized through artificial intelligence (AI)-based models for enhanced recovery of *Ocimum basilicum* L. essential oil (OBEO). The NaDES mixture was prepared at various compositional ratios and customized through statistical tools of RSM as well as AI-based models (ANN) to optimize the recovery and chemical composition of OBEO. The physicochemical properties of the prepared NaDES were studied at 5–50% molar ratios of water, which revealed a pH drift of 1–2.5, a viscosity of 1244–5.21 mPa.s, and a density of 1.55–1.22 g/cm^3^. The maximum yield of OBEO (41.00 ± 0.27 mL/Kg of fresh aerial material) was obtained with the extracting solvent containing 31.6% NaDES applied with 7.18 mL/g solvent-to-solid ratio at 70 °C and 210 min of distillation time. Interestingly, the chemical composition of OBEO obtained through the test protocol was more diverse in chemical nature in comparison to the control (hydrodistillation) when studied through GC-MS analysis. Moreover, NaDES-based OBEO demonstrated potent broad-spectrum antimicrobial activity, antioxidant activity (90.36% inhibition of DPPH^⸰^), and notable mosquito repellent activity (RC_50_ = 407 µL). These findings indicate that Glucose–Citric Acid-based NaDES in combination with water can distill OBEO more efficiently for its application in food and pharmaceutical industries.

## 1. Introduction

In recent times, there has been increasing interest in medicinal and aromatic plants as potential natural sources of bioactive substances owing to their strong antioxidant, antimicrobial, anti-inflammatory and insecticidal activities [1]. Plant species such as *Rosmarinus officinalis* (Rosemary), *Thymus vulgaris* (Thyme), *Origanum vulgare* (Oregano), and *Camellia sinensis* (Green Tea) have gained great interest owing to the presence of high quantities of phenols and essential oils [2]. Among these, one such promising plant is *Ocimum basilicum* (Basil) owing to the high yield of essential oil and rich phytochemical contents, which includes linalool, estragole, eugenol, rosmarinic acid, and flavonoids [3,4]. *Ocimum basilicum* L., commonly known as great basil, stands among the cultivated herbs of the *Lamiaceae* family [5]. The great basil (*O. basilicum*), as a whole plant or its leaves, is famous for its recreational, culinary, and traditional medicinal uses across Asia, the Middle East, and the Mediterranean region [6]. Studies have revealed that *O. basilicum* and its derived products possess antioxidant, antimicrobial, and insect-repellent properties [7], which could be associated with the presence of volatile phytochemicals like linalool, estragole, methyl chavicol, eugenol, and many others [8,9]. In previous studies, techniques like conventional hydrodistillation, steam distillation, solvent extraction, ultrasonic-assisted extraction, and microwave-assisted extraction have been applied for the extraction of the essential oil of *Ocimum basilicum* [10]. Nevertheless, these procedures are often linked to some drawbacks, including low extraction efficiency, thermal degradation of volatile components, residual solvents, or high operational costs [11,12]. *O. basilicum* essential oil (OBEO), which is obtained via hydrodistillation (HD), is particularly valued for its higher anti-inflammatory, antimicrobial, and antioxidant properties [13]. Malaria, a life-threatening disease that remains prevalent in tropical regions, poses a heavy burden on healthcare facilities, particularly affecting minors. Dengue, a similar but more serious infection, also spreads through mosquito bites [14].

Several studies showed that OBEO produced via HD becomes rich in estragole, linalool, and 1,8-cineol, which offer specific aromas that work as repellents against mosquitoes and other insects, as well as promising insecticidal properties [14]. However, the medicinal and commercial properties of OBEO are very dependent on the extraction protocol, whose development and optimization have been a challenging task. The frequently adopted hydro/steam distillation strategies are expedient, but these techniques offer limited recovery rates [15]. HD is frequently carried out using a Clevenger apparatus whose geometry affects the quantity, fragrance, and quality of essential oils [16,17]. A sustainable alternative to HD, supercritical fluid-based technologies, cannot be scaled up due to the exceptionally high capital cost. In addition, non-invasive solvent extraction involves the production of essential oil contaminated with toxic organic residues of methanol, chloroform, ethyl acetate, acetone, and *n*-hexane [18].

It has been widely accepted that deep eutectic mixtures possess higher heat capacities and non-volatile features, owing to the presence of strong intermolecular attractions [19]. Deep eutectic solvents (DESs) have recently emerged as a non-volatile and greener alternative to conventional solvents [20]. DESs offer a new model in green chemistry, and they can exist in millions of compositional combinations and ratio between hydrogen bond donor (HBD) and hydrogen bond acceptor (HBA) species. DESs are easy to prepare, inexpensive, biodegradable solvents with tunable polarity and solubility [21]. When DESs are prepared from a natural food-grade combination of compounds such as sugars, organic acids, and water, they are often referred to as a natural deep eutectic solvent (NaDES) [22]. Besides being thermodynamically more stable and generally regarded as safe (GRAS), many combinations of NaDES have been shown to disrupt the cell wall through different interactions, including hydrogen bonding and ionic interactions, which facilitate the release of bioactive compounds from plants [21].

Although these NaDES have been widely used in different plant extraction processes, there are few reported studies on their use in the distillation of OBEO. Previous research work focused on liquid/liquid, ultrasound, and enzyme-assisted extraction rather than integrating NaDES in the HD process. Meanwhile, our understanding regarding the formation of NaDES (low melting liquids) suggests that these liquids can retain more heat at a temperature to accelerate the evaporation of aromatic compounds and mass transfer. A careful review of previous research work indicates that no study has been undertaken to extract OBEO while applying NADES_HD. As such, we herein tested the use of glucose/citric acid (1:1) NaDES in combination as a less volatile and GRAS solvent for the distillation of OBEO. There were several other motives to consider glucose/citric acid-based NaDES, like its facile synthesis, hydrogen bonding ability in all ratios, chemical and thermal stability, cell wall macerating potential, and its prominent GRAS nature [23]. To cope with limited mass transfer due to the higher viscosity of pure NaDES, water was used as a modifier. The factors affecting OBEO recovery during NaDES distillation (i.e., amount of NaDES, solvent-to-solid ratio, distillation temperature, and distillation time) were tested at various levels. In addition to essential oil yield optimization, we tested the OBEO obtained via NaDES-based distillation for its antioxidant, antimicrobial, and mosquito-repellent activities. The outcomes of the present study provide a new framework for efficient and sustainable recovery of aroma compounds while applying a non-volatile and GRAS distillation green solvent.

## 2. Results

### 2.1. FT-IR Characterization of NaDES

**Figure 1:**
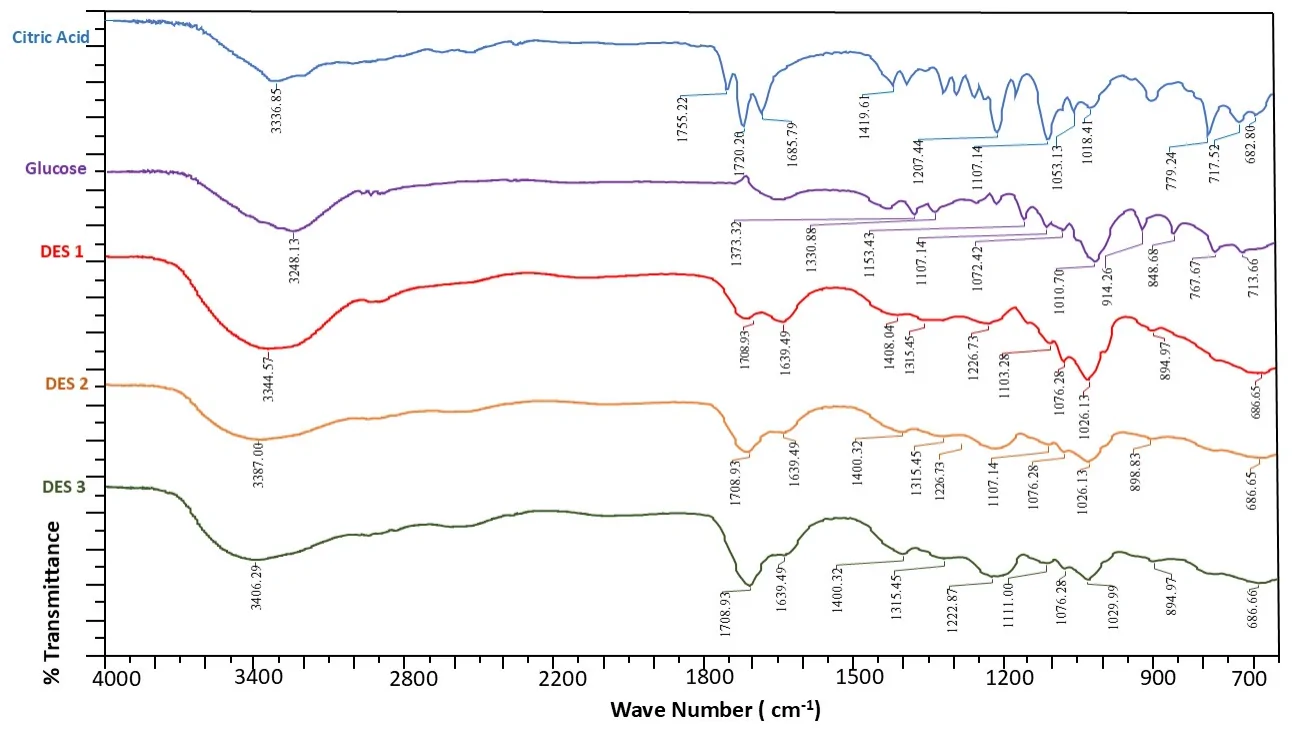
A comparison of Fourier Transformation Infrared (FT-IR) spectra of glucose (HBA), citric acid (HBD) and NaDES.

Figure 1 presents the FT-IR spectra of synthesized NaDES, glucose, and citric acid. It is obvious from the spectrum that the former produces three prominent transition frequencies with a broader peak at 3500–3200 cm^−1^, multiple bands at 1750–1700 cm^−1^, and peculiar behavior at 1200–1050 cm^−1^, probably associated with stretching vibrations of hydroxyl groups (OH), carbonyl group (C-O), and bending vibrations of the C-O-H group, respectively. Likewise, glucose absorbs in more intense and broader regions of 3600–3000 cm^−1^ due to the presence of multiple OH groups [24], followed by characteristic peaks at 914 and 848 cm^−1^, which are characteristic of α-D-glucose [25].

### 2.2. Viscosity and Density of NaDES

**Figure 2:**
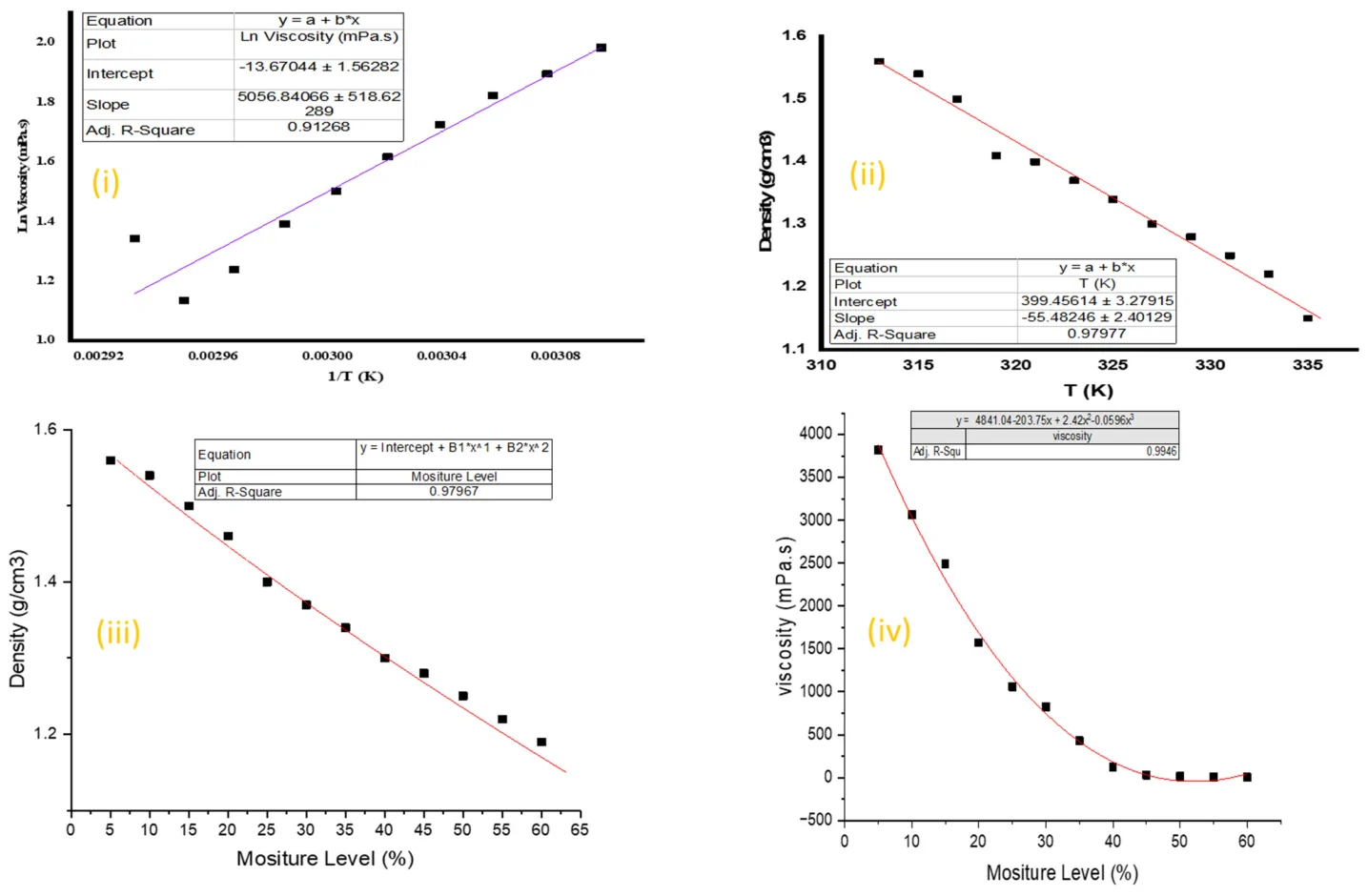
Effect of temperature on (**i**) viscosity and (**ii**) density, and effect of water level on (**iii**) density and (**iv**) viscosity.

The data plotted in Figure 2i–iv indicate the variation in viscosity and density of synthesized NaDES in response to change in temperature and water level.

### 2.3. One-Factor-at-a-Time (OFAT) Analysis

**Figure 3:**
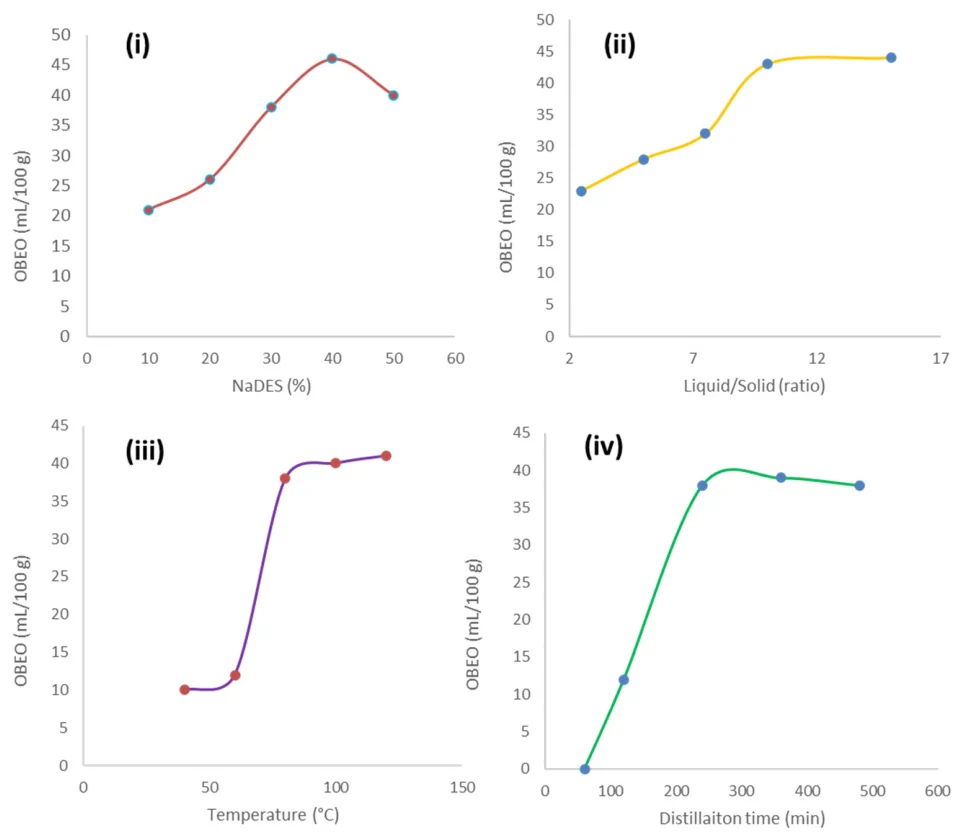
The classical trials to study One-Factor-at-a-Time (OFAT) for suitable selection of (**i**) %NaDES, (**ii**) solvent-to-solid ratio, (**iii**) temperature and (**iv**) time, for enhanced recovery of OBEO.

The data plotted in Figure 3i–iv illustrate how the quantity of OBEO (mL) recovered varied with the change in distillation conditions.

### 2.4. ANOVA and Fit Statistics

**Figure 4:**
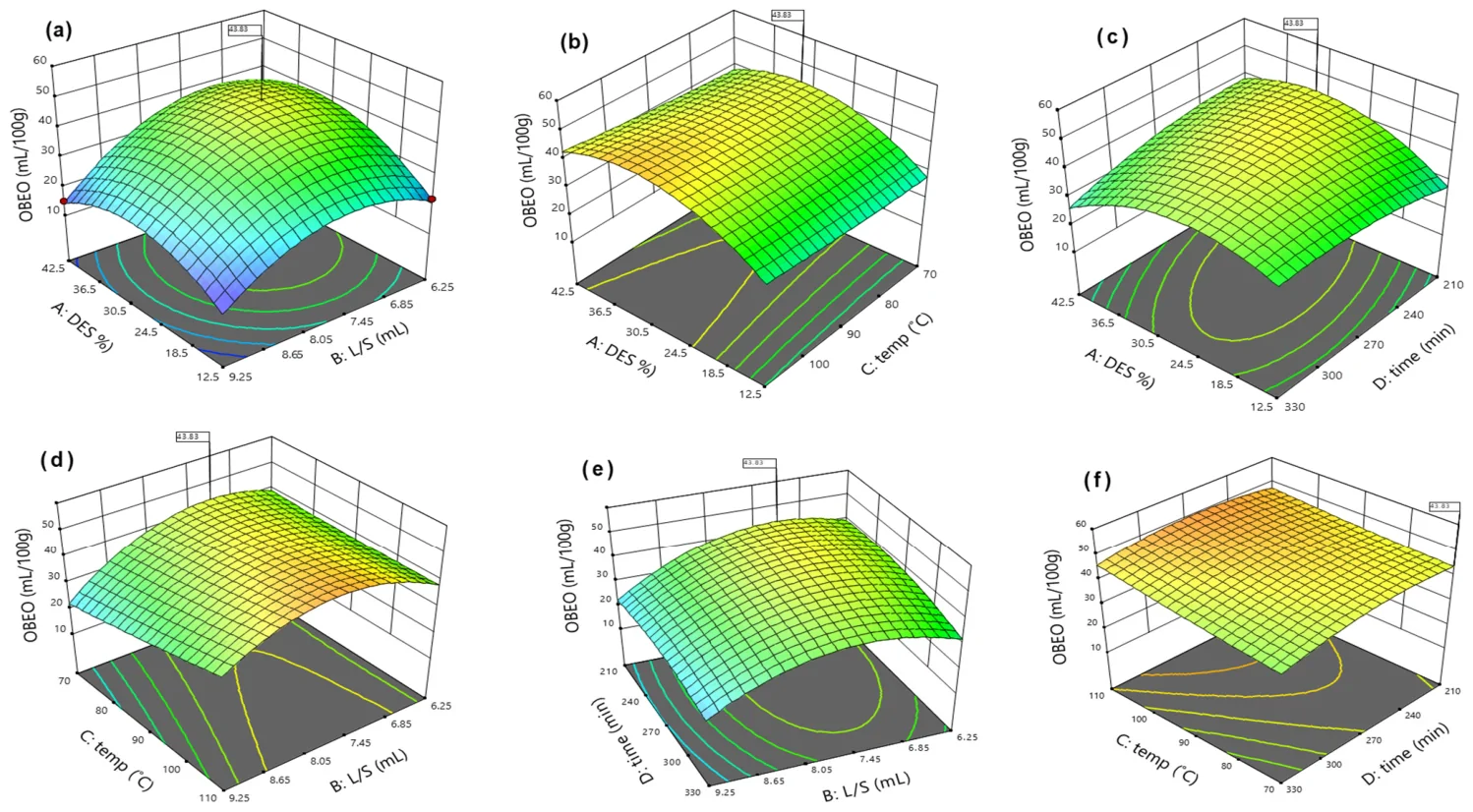
Three-dimensional plots showing the combined effects of process variables (**a**) DES (%) and L/S (ratio), (**b**) DES (%) and Temperature, (**c**) DES (%) and time, (**d**) Temperature and L/S ratio, (**e**) time and L/S ratio, and (**f**) Temperature and time on essential oil yield, highlighting quadratic trends and optimal OBEO region.

The analysis of variance (ANOVA) and F-statistics for OBEO yield obtained under various conditions have been presented in Table 1 whereas the interaction between extraction variable can be better understood from Figure 4.

### 2.5. Artificial Neural Network Analysis

**Figure 5:**
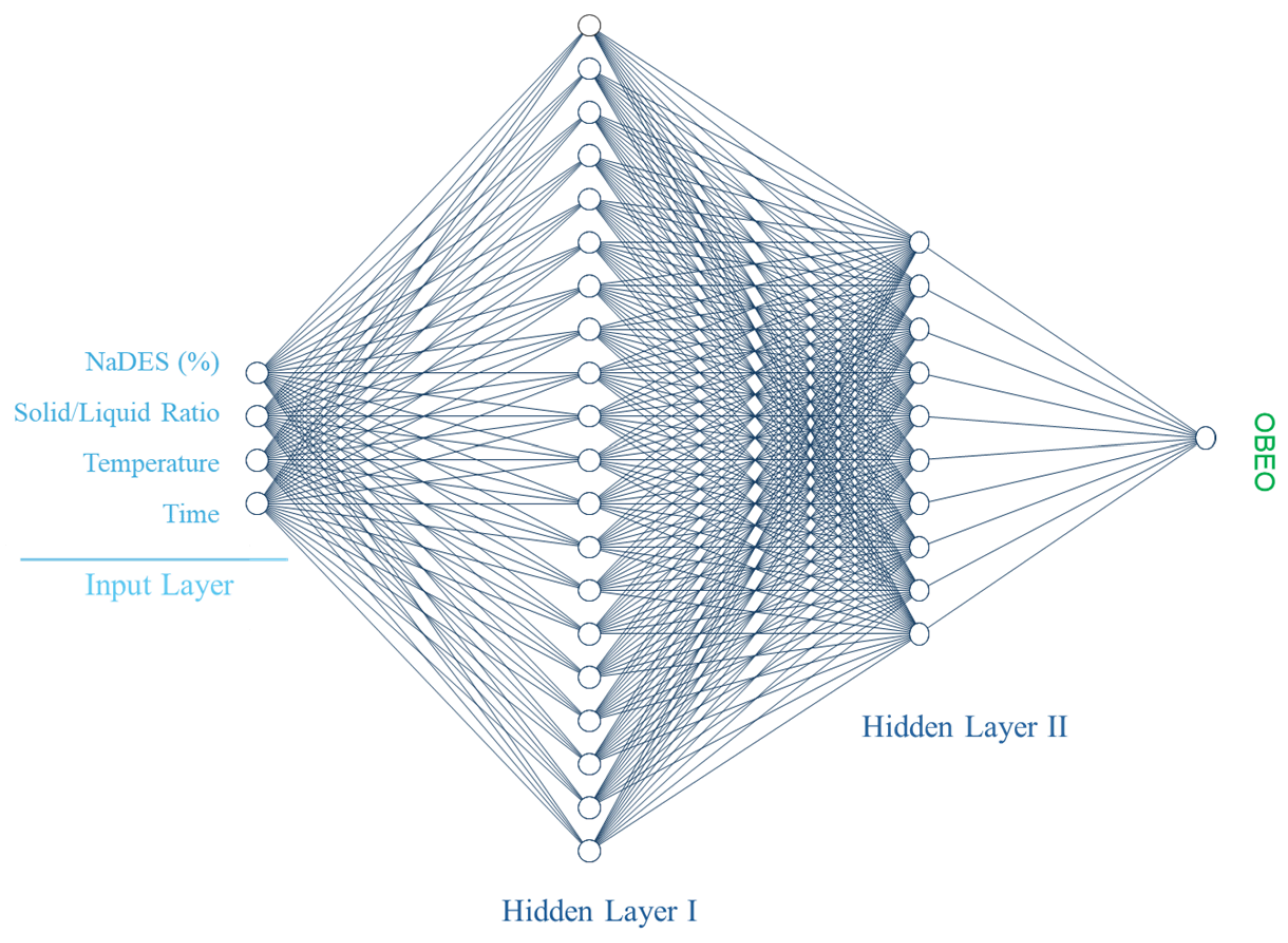
Artificial neural networking representation of four input, two hidden, and one output layer to predict OBEO yield (mL/g of plant material).

The ANN model outputs, optimized equations, including the 20-neuron hidden layer formulations, are illustrated in Figure 5.

### 2.6. Experimental Validations

Figure S1 provides a ramp of the most suitable solution for NaDES conditions, which comprises NaDES 31.6%, solvent-to-solid ratio of 7.18 mL/g, temperature of 70 °C, and distillation time of 210 min. Likewise, the results in Table S2 show the comparison of predictive accuracy of RSM and ANN for essential oil yield optimization.

### 2.7. Phytochemical Profile of OBEO

OBEO obtained using the developed NaDES-assisted distillation protocol and conventional hydrodistillation (HD, control) was analyzed by gas chromatography–mass spectrometry (GC–MS), and the corresponding chromatograms are presented in Figures S2 and S4. Compound assignments were made by comparing the acquired EI mass spectra with spectra available in the NIST mass spectral library, together with their chromatographic retention times and library similarity indices as shown in Figure S5. Since experimental retention indices and authentic reference standards were not available in the present study, the reported compounds are considered tentatively identified rather than unequivocally confirmed. The GC–MS profiles indicated differences in the relative chemical composition of OBEO obtained by the two extraction approaches, as summarized in Table 2 and Table S3**.** Particular caution was applied to the interpretation of less characteristic constituents, including isopropyl myristate and fatty-acid methyl esters, and these assignments should therefore be regarded as provisional pending confirmation by retention-index analysis and/or authentic standards.

### 2.8. Biological Activities of OBEO

#### 2.8.1. Antimicrobial Studies

**Figure 6:**
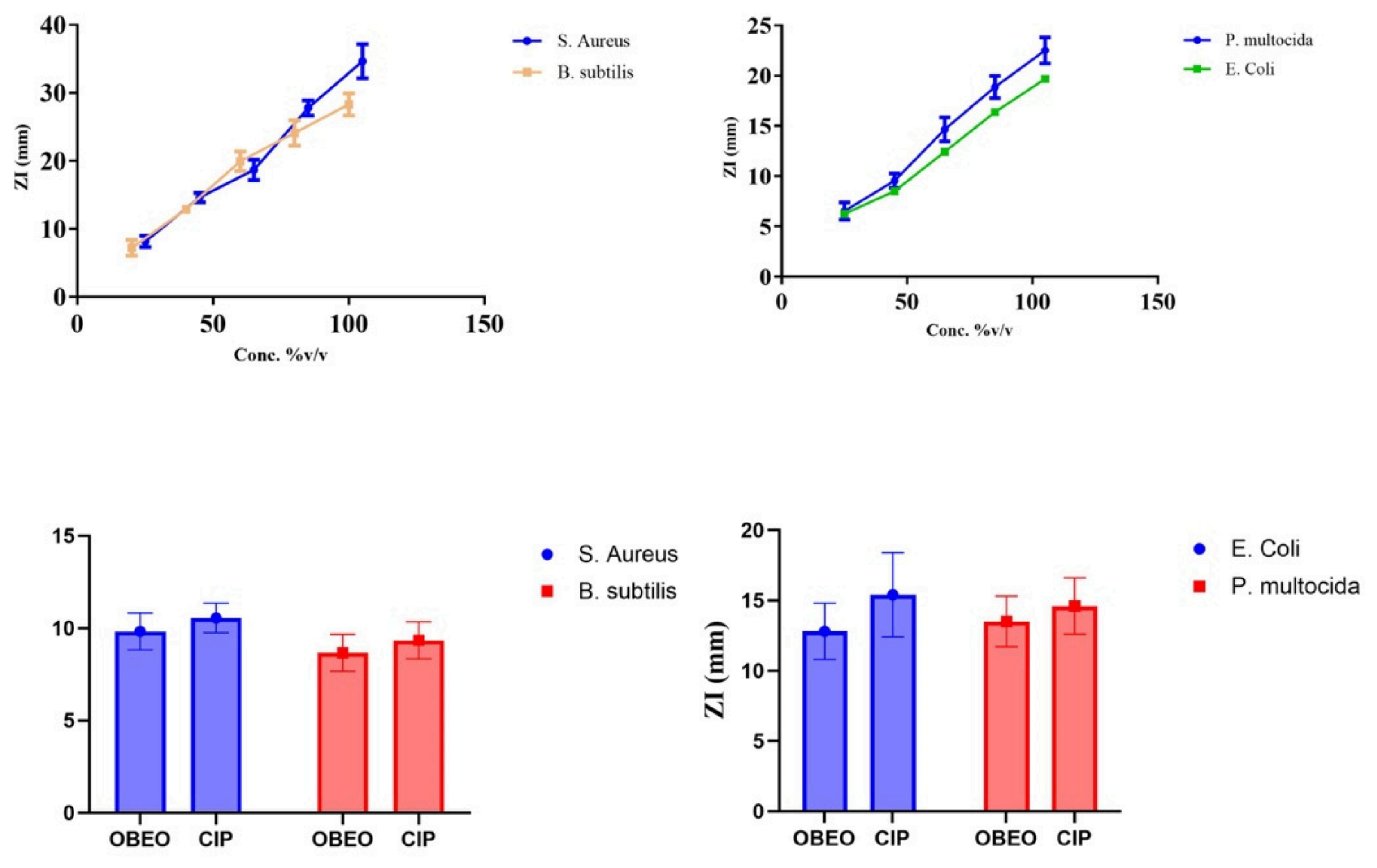
Dose-dependent response of OBEO against selected bacterial strains, bar graph represents the zone of inhibition of the standard drug ciprofloxacin against each strain.

The antimicrobial activity of the OBEO obtained by NaDES was evaluated against various bacterial strains, and the results are given in Figure 6. OBEO showed increasing antimicrobial effect in a dose-dependent way on all the tested bacterial strains. *Staphylococcus aureus* is the most sensitive, while the least inhibition was seen in the case of Gram-negative bacteria (*E. coli* and *P. multocida*). This may be attributed to the protective outer membrane of Gram-negative organisms [10].

#### 2.8.2. Antioxidant Evaluation

**Figure 7:**
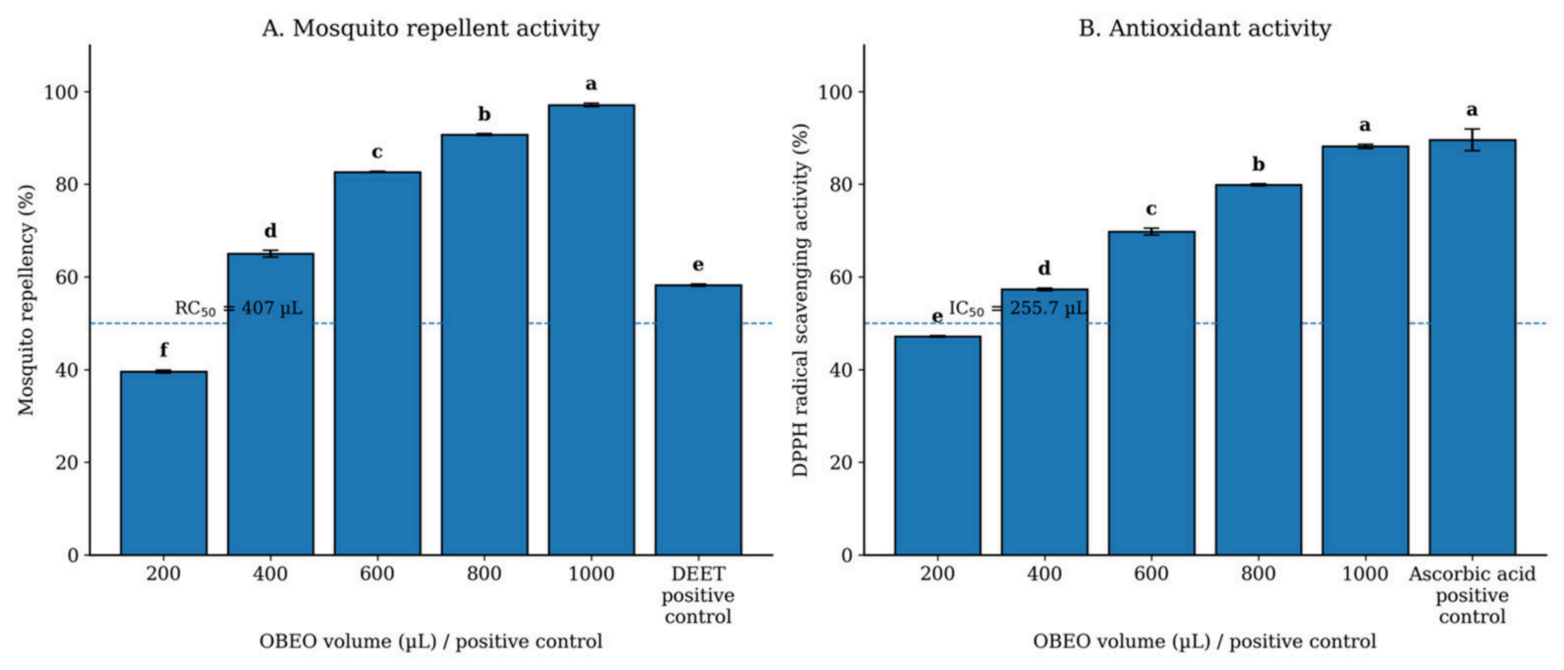
Mosquito-repellent and antioxidant activities of OBEO obtained via NaDES-assisted distillation. Values are mean ± SD, *n* = 3. Different letters indicate significant differences at *p* < 0.05.

The antioxidant potential of OBEO prepared using NaDES was investigated using the DPPH (radical scavenging) assay. Different concentrations of OBEO obtained under optimum conditions of NaDES-D, ranging from 50 to 250 µL, were tested. Clear dose-dependent free-radical scavenging activities were observed as shown in Figure 7B.

#### 2.8.3. Mosquito-Repellent Activity

The repellent activity of OBEO extracted in NaDES was assessed using the wooden box assay against adult mosquitoes. Cotton swabs containing OBEO at different concentrations (200 µL to 1000 µL) were tested, and the percentage repellency is shown in Figure 7.

## 3. Discussion

### 3.1. Synthesis and Characterization of NaDES

Natural deep eutectic solvent (NaDES) was prepared comprising an equimolar ratio of glucose/citric acid/water (1:1:1). The reactant mixture was kept at 80 °C under constant stirring in a vacuum flask until a homogeneous and transparent mixture was formed. The NaDES product was collected and characterized for physiochemical and structural properties to investigate the presence of possible interactions between hydrogen bond donor (HBD) and hydrogen bond acceptor (HBA). Physiochemical properties of NaDES including density, viscosity, refractive index, and the possible effect of temperature and water level on these properties were investigated. Chemical characterization of NaDES was carried out through FT-IR in comparison to spectra of citric acid, glucose, and their eutectic supramolecule (NaDES) as given in Figure 1.

The formation of NaDES involves the formation of hydrogen bonds between citric acid (HBD) and glucose (HBD/HBA), which can be confirmed through the very broad red shift observed at 3344 cm^−1^. The peak broadening can be attributed to the fixation of glucose OH groups and, similarly, the shifting of the carbonyl band of citric acid towards 1708 cm^−1^ indicates complexation in NaDES due to formation of a hydrogen bond between carbonyl oxygen of citric acid and hydrogen of glucose (here glucose works as HBD). In other words, it is obvious from the FT-IR data that, during the formation of glucose/citric acid-based NaDES, glucose works as HBD as well as HBA.

### 3.2. Viscosity and Density of NaDES

The viscosity and density of solvents play a crucial role during extraction of analyte of interest from plant samples [26]. The density of NaDES is affected by various input variables described in Figure 2; Figure 2i–iv indicates variation in these two physiochemical variables with change in temperature and water level estimated through Equation (4). Relative higher viscosities of NaDES (glucose/citric acid) from pure individual solutions indicate the presence of strong hydrogen bonding in NaDES. However, with the increase in temperature, intermolecular spacing and molecular mobility increase, leading to slightly decreased hydrogen bonding. Meanwhile, a higher slope and activation energy of a graph (Figure 2i) plotted between viscosity and the inverse of temperature indicate the presence of ordered hydrogen bonding with very high activation energy (≥42 kj/mol) following the Arrhenius model. The adjusted R^2^ ≥ 0.95 indicates good agreement between observed and predicted data, ruling out anomalies in hydrogen bond network within the tested temperature range. A similar kind of behavior has been previously observed by [27] for citric acid and sucrose (1:1) based NaDES.

(1) $$\eta =\eta_{0}e^{\frac{E_{a}}{RT}}\;$$

The variation in viscosity with change in water level was interesting and somehow difficult to justify, as manifested in Figure 2iv. For the smaller amounts of water level (i.e., 1–10%), NaDES remains intact while water molecules contribute towards free volume and exponentially reduce the viscosity of NaDES. Any further additions of water start lubricating glucose and citric acid that mask the strength of hydrogen bonding among HBD and HBA. Instead, both HBA and HBD become hydrated and lead to an abrupt decrease in forces of attraction between HBD and HBA and a decline in viscosity following a higher-order polynomial equation as shown in Figure 2iv. However, when water contribution reaches ≥20%, its dielectric behavior becomes visual between glucose and citric acid molecules to make the liquid flow following Newtonian flow dynamics. Finally, dilution above 70% breaks the interaction between HBD and HBA and makes NaDES a dilute solution or independent mixture of glucose and citric acid. The density of glucose/citric acid (1:1)-based NaDES decreases linearly with the increase in temperature and water level, as obvious from the data plotted in Figure 2ii,iii. It could be further mentioned here that R^2^ ≥ 0.95 indicates that there is good agreement between the observed decrease in density with the increase in temperature as per Equation (1) and dilution ratio, which implies that prepared NaDES does not undergo any phase transitions and is free from any structural anomaly.

### 3.3. NaDES-Based Distillation of OBEO

The OFAT analysis (Figure 3) revealed that the recovery of OBEO from fresh leaves during NaDES-based distillation varied with changes in NaDES/water ratio, solvent to substrate ratio, extraction time, and temperature. The most prominent and interesting effect on recovery of OBEO was observed for NaDES/water ratio. Explicitly speaking, the amount of glucose/citric acid (1:1)-based NaDES below 20% could not enhance the recovery of OBEO. However, an increase in OBEO level was observed with the increase in DES ratio. The increase in OBEO can be attributed to the decrease in viscosity and density and the increase in heat capacity of NaDES with the increase in water level. It is obvious from the data presented in Figure 3i that, above 50% of NaDES ratio, there was a decline in OBEO recovery rates. This decline might have happened due to the decomposition of NaDES with water level of >50%. At this water level, it works only as a dilute solution which has low heat capacity. Therefore, in the case of deep eutectic liquid-based hydrodistillation, the thermodynamics and mass transfer dynamics play opposite roles, and we need to find an optimum condition regarding their use as a suitable extraction solvent. Following the NaDES amount, an increase in liquid/solid ratio improved the OBEO recovery, but the most significant increase was observed for liquid to solid ratio between 10 and 20% (Figure 3ii), which is supported by the literature [28]. Similarly, the extraction time increases the yield by 40–48%, but prolonged extraction could not offer parallel effects. In contrast, an increase in temperature increased the recovery of OBEO from 41 to 55%, suggesting an enhanced heat capacity of distillation medium, while a decrease in viscosity and density led to better mass transfer for NaDES-based distillation. Overall, the data presented in Figure 3i–iv demand a more comprehensive design of experiment (DoE) for the process optimization to enhance the OBEO recovery.

### 3.4. Process Optimization for NaDES Distillation of OBEO

Optimization of the factors screened during classical trials was undertaken following 5^4^ factorial design (four factors each at five levels) in a central composite design (CCD) of response surface methodology (RSM). The four factors are (A) NaDES percentage, (B) solvent-to-solid ratio, (C) temperature, and (D) extraction time. Following the data observed during initial trials, selected factors like NaDES percentage, solvent-to-solid ratio, distillation temperature, and time were tested in the range of 5–50%, 5.5–10 mL/g, 70–120 °C, and 180–360 min, respectively, as assembled in Table S1. The highest yield of OBEO (55 mL/Kg) was observed at run no. 1 of Table S1**,** which involves the conditions of NaDES comprising 27.5% NaDES, 7.75 mL/g solvent to feed ratio, 120 °C, and 270 min distillation time, indicating that a moderately high temperature and balanced solvent ratio promoted extraction efficiency. In contrast, factorial runs at extreme conditions, such as Run 17 (42.5% NaDES, 9.25 mL/g, 70 °C, and 210 min) and Run 7 (27.5% NaDES, 10 mL/g, 90 °C, and 270 min) resulted in lower OBEO yields, suggesting that excessive solvent or suboptimal temperature suppressed extraction performance. It can be concluded that NaDES mixed with water in ratios ≤50% in combination with higher solvent ratio/feed ratio, extraction temperature and time exerts a strong synergistic effect, while extreme conditions tend to reduce efficiency. The ANOVA and fit statistics were utilized for the determination of model significance and are given in Table 1. Statistical tools such as RSM are particularly useful to optimize the complex interaction with a minimum number of experiments. RSM has an advantage over the OFAT strategy in that it can successfully explain and interpret the independent variables when their first order and higher interactions become dominant. ANN, on the other hand, can be used to check the non-linear complex relationships between input variables and responses.

Table 1 shows a high degree of statistical significance, showing the model’s F-value of 69.69 and the corresponding *p*-value (<0.0001). This indicates that the model is highly effective at explaining the variation in the response and is not a result of random chance. The high R^2^ value of 0.9939 shows the good agreement between observed and predicted values. The Lack of Fit F-value of 0.6001 with a *p*-value of 0.5917 confirms the model is fit and can effectively represent the experimental data. The linear term C and the quadratic terms A^2^ and B^2^ (*p* ≤ 0.0001) show the most pronounced effect on yield of OBEO.

The effects of independent variables were studied for the yield of OBEO using NaDES under various conditions through RSM. 3D response graphs are presented in Figure 4. Figure 4a illustrates the influence of molar ratio of NaDES/water and solvent-to-solid ratio, where a well-defined central plateau is observed, reflecting a strong quadratic effect of the independent variables on yield. Both high and low levels of NaDES concentration and solvent ratio were unfavorable for OBEO extraction. According to the best of our literature survey, no study has been undertaken to extract OBEO while applying DES-HD. However, the conventional extraction of essential oil from *Pelargonium* species was affected by solvent concentration and solid to liquid ratios [29]. The high NaDES concentration increases the viscosity of distillation mixture, which reduces the mass transfer rates and amount of energy transferred to entrapped volatile compounds. On the flip side, the presence of solvents having low boiling points decreases the yield. The solvent-to-solid ratio is more pronounced when compared with temperature. The dome-shaped curvature in Figure 4c shows the interaction of NaDES % and extraction time, where yields decline beyond the optimum due to reduced solubility at higher NaDES concentrations. Figure 4d,e depict the effect of temperature and the solvent ratio and temperature with solvent-to-solid ratio, respectively, both showing quadratic trends. These results demonstrate the importance of optimization in essential oil extraction, as high temperature and solvent ratio decrease the yield due to dilution and thermal degradation. Figure 4f shows a nearly planar inclination with a gradual slope, indicating minimal quadratic contribution. This suggests that this parameter combination has a comparatively less critical role in determining yield. Equation (5) shows the model equation for the yield of essential oil extracted using NaDES-HD. The temperature and solvent ratio have a positive impact on essential oil yield.

Y_(yield)_ = 47.83 − 2.33 B + 4.40 C − 2.75 AB − 4.58 AD + 4.00 BC − 9.78 A^2^ − 11.34 B^2^ − 3.11 D^2^ (2)

In this study, RSM and ANN were employed for modeling and prediction of OBEO extraction yield. RSM served as the primary experimental design and optimization framework, whereas ANN was applied as a complementary nonlinear predictive approach to evaluate the relationship between process variables and extraction yield. The ANN was trained using the Levenberg–Marquardt algorithm by minimizing the mean squared error (MSE) through iterative adjustment of network weights and biases. Model performance was evaluated using the correlation coefficient (R^2^) and MSE. The ANN produced R^2^ values of 0.99078, 0.99957, and 0.99977 for the training, testing, and validation subsets, respectively, with an overall R value of 0.99428. These values indicate a close correlation between the experimentally observed and ANN-predicted responses within the present dataset. However, considering the limited number of experimental runs used for ANN development, these performance indices were interpreted cautiously and were not considered evidence of broader model generalizability or unequivocal superiority of ANN over RSM. The minimum validation MSE (0.20387) was observed at epoch 0, indicating that subsequent training iterations do not further improve the validation performance. Therefore, this observation was not interpreted as evidence of enhanced ANN generalization. At epoch 4, the gradient reached 5.2468 × 10^−13^. Accordingly, the ANN results were considered together with the RSM predictions and subsequent experimental validation of the optimized conditions rather than being used as an independent basis for establishing the optimum extraction conditions.

### 3.5. Validation of Optimum Conditions

According to Figure S1, the optimized conditions were predicted to produce 43.83 mL of OBEO/kg of *O. basilicum* leaves. A new set of validation experiments was subsequently performed under these conditions, resulting in an experimentally observed OBEO yield of 41.00 ± 0.27 mL/kg. Table S2 compares the experimental and predicted yields obtained using RSM and ANN in terms of %RSD, percentage prediction error (PPE), RMSE, R^2^, and %AAD. Both modeling approaches showed reasonable agreement between the predicted and experimentally observed responses. Within the present experimental dataset, ANN produced lower %RSD (2.08 vs. 4.72), PPE (2.90 vs. 6.90), RMSE (0.65 vs. 0.85), and %AAD (0.91 vs. 2.55), together with a slightly higher R^2^ value (0.9977 vs. 0.9954), compared with RSM. However, considering the limited number of experimental runs used for ANN development, these performance indices should be interpreted cautiously and should not be considered evidence of broader model generalizability or unequivocal superiority of ANN over RSM. In the present study, RSM served as the primary experimental design and optimization framework, whereas ANN was employed as a complementary nonlinear predictive approach. The subsequent experimental validation showed close agreement between the predicted and observed OBEO yields, providing practical support for the selected optimum conditions. Thus, the optimized conditions were established on the combined basis of the CCD–RSM experimental design, predictive modeling, and experimental validation rather than on ANN performance alone.

### 3.6. GC-MS Analysis of OBEO

The presence of diverse natured volatile components mainly comprised of sesquiterpenes, monoterpenes, and fatty acids is observed in OBEO extracted using NaDES (details are provided in Table 2). Although a large number of components have been observed, a total of 13 compounds are reported, indicating reliable identification based on retention time, mass spectra, chemical structure, and similarity index (>85) with NIST Library, as described in Figure S3. Chromatographic analysis revealed that linalool, an oxygenated monoterpene, detected at a retention time (RT) of 8.74 min, is observed as most abundant, representing 24.32% of the total chromatographic area. Linalool is widely reported as one of the major components in essential oil of basil and is commonly known for its biological activities like antioxidant, antimicrobial, anti-inflammatory, etc. [30]. Another major component, estragole (4-allylanisole), was detected at RT of 10.34 min, accounting for 13.68% of total chromatographic area. This component is also frequently reported as basil chemotypes responsible for characteristic aroma and associated with insecticidal and antimicrobial properties [31]. Among the sesquiterpene family, caryophyllene identified at RT of 16.09 min is observed as relatively abundant, accounting for about 6%, mostly contributing to enhanced antioxidant and antimicrobial properties. In addition to terpenoids, various fatty acids were also detected, among which some were minor in relative abundance but may significantly contribute to the biological activity and physiochemical properties of OBEO. However, GC-MS chromatographic results of OBEO (control) revealed that linalool is highly abundant (41.66%) while estragole is absent. Similarly, T-Cadinol and 2-Norpinene, among several other compounds, were not observed in EO (control) while present in EO (NaDES), as given in Table 2 and Table S3. Overall, GC-MS profiles indicate that basil essential oil extracted using NaDES is rich in bioactive and chemically complex profile that may contribute to the overall therapeutic and insecticidal properties. This difference in composition, as well as yield, suggests that proposed extraction protocol can be efficiently viable for economical and greener EO extraction with customized chemical compositions.

Several other studies have also revealed a chemotype variation of basil essential oil depending on extraction techniques. The presence of dominant components of linalool and estragole along with caryophyllene and sesquiterpenes in the treated sample of the present study is consistent with previous reports while a mere difference in compositional proportion. For example, steam distillation of basil showed estragole (70–79%) as a major component, followed by linalool (11–13%), while there were traces of other constituents [32]. Similarly, chemical composition of EO obtained through hydrodistillation is observed to be dominant with estragole (≈85%) [19], while EO obtained through microwave-assisted extraction contains estragole at merely about 10% [33]. In comparison to these reports, EO obtained using NaDES showed a very balanced level of both linalool and estragole along with a diverse nature of fatty acids and other terpenoids. This variation comparison further suggests that the extraction protocol outlined in the present study may also influence the recovery as well as the composition of volatile constituents, potentially affecting the biological activity and other physiochemical properties. The NaDES-based extract may reflect strong antimicrobial, antioxidant, and mosquito-repellent activities that can be explained by the high abundance of linalool (24.32%), a well-known antimicrobial and antioxidant monoterpene, and estragole (13.68%), which exerts potent antimicrobial and insecticidal properties. Moreover, caryophyllene, which is known for its antioxidant and antimicrobial activities along with other minor components, including limonene, eucalyptol, T-cadinol, and fatty acid derivatives, may enhance the essential oil’s overall biological efficacy.

### 3.7. Antimicrobial Activity of OBEO

Interestingly, a dose-dependent response was observed for inhibition of zone against each bacterial strain studied, which showed broad-spectrum antibiotic nature of test material. The antimicrobial effect of OBEO observed at (60% *v*/*v*) concentration was comparable with that of 1000 ppm standard antibiotic drug ciprofloxacin, as given in Figure 6. The negative controls (distilled water, ethanol, and DMSO) showed no inhibition zone, confirming that the antimicrobial activity was caused by the extracted *O. basilicum* essential oil. These results demonstrate the potent antimicrobial efficacy of the essential oil obtained using NaDES. Moreover, the minimum inhibitory concentration (MIC) of extraction showed good activity against *P. multocida* of MIC value of 3.125% *v*/*v*, as given in Table S4. In comparison, Adigüzel and colleagues reported only limited antimicrobial activity (6–10%) against various bacterial strains when preparing extracts with conventional techniques by using solvents like hexane, methanol, and ethanol [34]. This apparent difference in bioactivity highlights the superior performance of NaDES as an efficient and green extraction technology, which not only minimizes the use of hazardous organic solvents but also enhances the recovery as well as diversity of constituents responsible for biological potential. This improved performance may also be associated with the diverse chemical nature of OBEO obtained through the developed protocol, which suggests that bioactivity can be improved through a customized extraction technique. These findings also open new opportunities regarding the use of NaDES as an efficient and environmentally sustainable alternative to conventional solvent-based methods for isolating natural antimicrobial compounds.

### 3.8. Antioxidant Activity of OBEO

Overall, 1000 µL of OBEO offers the highest inhibition (88.17 ± 0.43%) of free radicals, which demonstrates a strong radical scavenging activity. Likewise, the antimicrobial and antioxidant activities of the highest concentration were comparable with those observed using 200 µL of 100 ppm ascorbic acid. After careful literature review, no study has been undertaken to utilize NaDES-based distillation of essential oil. However, the antioxidant activity of OBEO was investigated by following the protocols of Qamar and colleagues and compared with standard reference material of ascorbic acid [35]. This demonstrates the potential of NaDES as a greener and more effective extraction medium for recovering volatile bioactive compounds with strong antioxidant capacity.

### 3.9. Mosquito-Repellent Activities

The data presented in Figure 7 illustrate that OBEO obtained under optimum conditions exhibited strong mosquito-repellent activities in a dose-dependent manner. The mosquito-repellent activity shown by OBEO at the highest tested concentration, i.e., 1000 µL was comparable with 100 µL of 200 ppm DEET (reference standard). Previously, Baba and colleagues reported the mosquito-repellent activity of OB leaves extracts prepared using hexane and repellent activity of OBEO obtained via NaDES in the present study demonstrated better repellent activity than OB leaves extracts prepared using hexane [36]. The hexane extract at 50% and 100% concentrations showed efficient repellent activity, but a high concentration is required for extended protection time. The OBEO produced via NaDES distillation demonstrated a strong, concentration-dependent increase in repellence even at relatively lower volumes. At 200 µL, OBEO achieved 39.57 ± 0.35% repellence, which increased steadily to 97.11 ± 0.35% at 1000 µL. This indicates that OBEO produced via NaDES can yield a half maximal repellency concentration (RC_50_) of 407 µL depending upon nonlinear regression analysis. These results prove that the NaDES-based distillation produces essential oil OBEO with stronger mosquito repellent activity. Overall, it was observed that NaDES enhances efficacy while reducing reliance on hazardous organic solvents.

The biological activities observed for NaDES extract could be attributed to balanced phytochemical composition in addition to improved extraction yield. Previous reports about extraction of OBEO demonstrated high abundance of selective compounds like linalool (43.5–48.4%) using microwave-assisted hydrodistillation [37] and estragole (up to 85%) by ultrasound-assisted hydrodistillation [19]. Although, linalool was also a major constituent in our study (24.32%) but comparatively lower than the literature, along with compositional balance with T-Cadinol, Caryophyllene, 4-Allylanisole, etc., that has been extensively linked to antioxidant, antimicrobial, and anti-inflammatory properties [38]. It has been shown that minor ingredients like eucalyptol and limonene [39] contribute to various antioxidant and antibacterial activities. Therefore, the improved biological performance reported in this study may be explained by the recovery of these extra chemicals by NaDES-assisted distillation.

## 4. Materials and Methods

### 4.1. Reagents Used

All the chemicals and reagents used during the whole experimental work of this study were of analytical grade and used as such without further purification. Citric acid (≥98.5%), glucose (≥97%), 2,2-diphenyl-1-picrylhydrazyl (DPPH) (≥98%), Dimethyl-sulfoxide (DMSO) (≥99%), ascorbic acid (≥98.6%), and ciprofloxacin (≥99.2%) were purchased from Sigma-Aldrich, USA. Nutrient broth (≥95%) and Muller Hinton agar (≥98%) were supplied by Thermo-Fisher Scientific, UK. All the bacterial strains (*Escherichia coli* and *Staphylococcus aureus* etc.) used in this study were collected from American Type Culture Collection (ATCC-10231). Deionized water and ethanol were supplied by Merck, Darmstadt, Germany. IR spectra of NaDES and its precursors were recorded through ATR-FT-IR IR Tracer-1800 (Shimadzu, Kyoto, Japan) by placing sample directly on Lense (ZnGeSe) and scanning at 4000–600 cm^−1^ with an average of 20 scans at a resolution factor 6. Specific density and viscosity of solvents and mixture solutions were measured with digital pycnometer and viscometer, respectively, at room temperature. Water contents of NaDES mixture were measured through Tiamo Karl Fisher (Metrohm, Herisau, Switzerland).

### 4.2. Sample Collection and Preparation

*Ocimum basilicum* L. plant was purchased from the nursery near the cantonment area, Lahore, Pakistan, and was identified by Dr. Tehreema Iftikhar (Botany Department, GCU, Lahore, Pakistan). For the botanical classification and identification of the plant, the specimen of sample material was submitted through voucher/reference # 1256 to Dr. Tehmeena Iftikhar, Professor at Government College University, Lahore, Department of Botany. Healthy leaves and flowers were carefully separated from the plant, washed, and blended with the NaDES-based extraction solvent mentioned in the subsequent section.

### 4.3. Preparation and Characterization of NaDES

**Figure 8:**
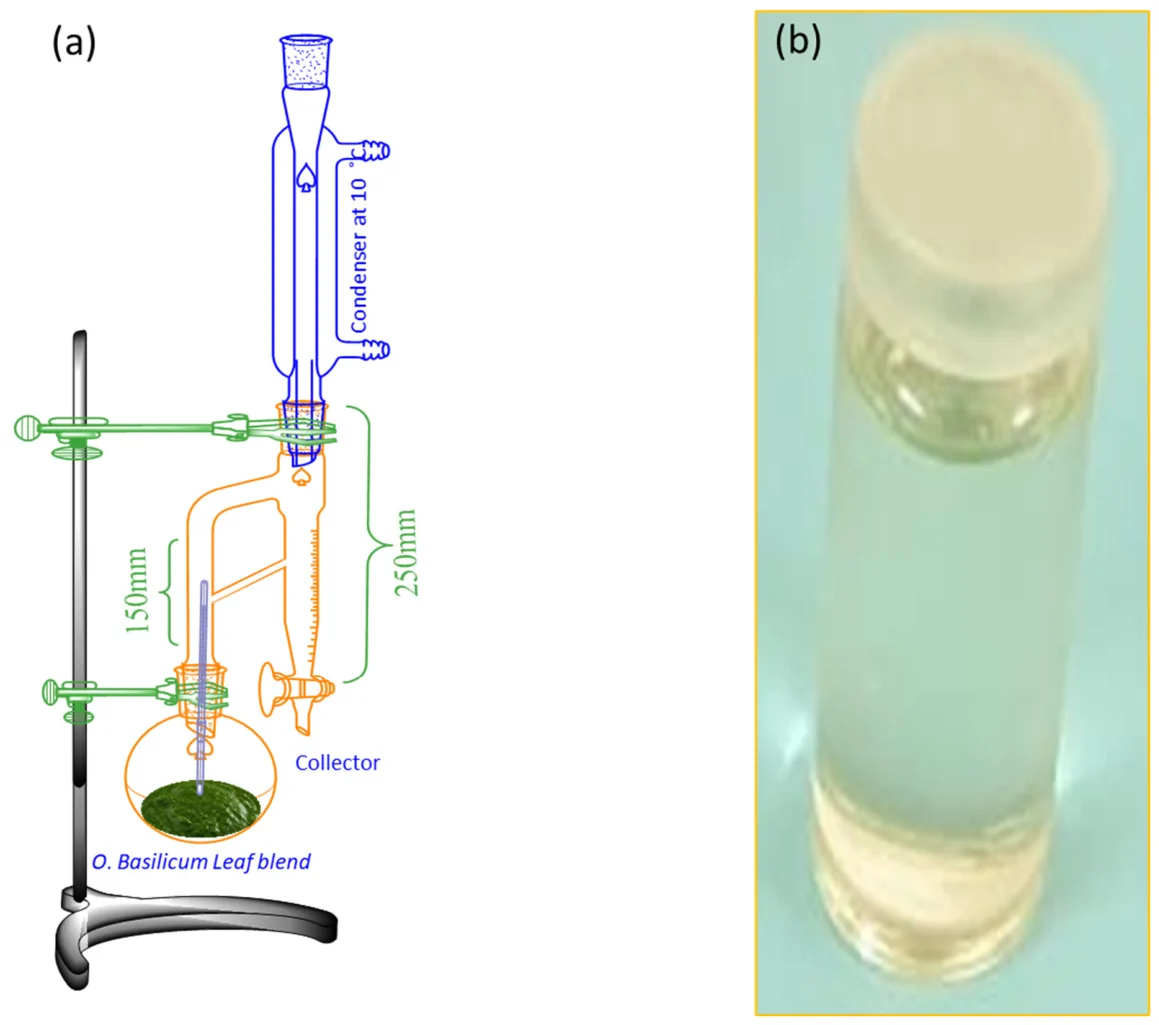
(**a**) The Clevenger apparatus used to distill *O. basilicum* leaf essential oil applying glucose/citric acid NaDES; (**b**) prepared NaDES mixture used to extract OBEO.

For the preparation of NaDES, equimolar amounts of citric acid (192 g), glucose (180 g), and 18 g of water were mixed in a 1.0 L vacuum flask. The contents were kept at 80 °C under constant stirring until a homogeneous and transparent mixture was formed, as shown in Figure 8b. This solution was further subjected to FT-IR, physicochemical evaluation, and Karl–Fischer titration to authenticate the formation of glucose–citric acid NaDES and % level of water [40].

### 4.4. NaDES–Water for the Distillation of O. basilicum Essential Oil (OBEO)

To collect OBEO, a mixture of *O. basilicum* leaves and the NaDES–water system was prepared in a 500 mL round-bottom flask and subjected to hydrodistillation using a Clevenger-type apparatus, as shown in Figure S1a. The distillation flask was heated using a temperature-controlled heating mantle. The temperatures investigated in the experimental design therefore represent the heating-mantle set-point temperatures rather than the actual temperature of the liquid or vapor inside the distillation flask. The NaDES content, liquid-to-solid ratio, and heating-mantle set-point temperature were varied according to the experimental conditions presented in Table 3. The Clevenger apparatus was connected to a condenser maintained at 10 ± 1 °C [41] to ensure efficient condensation and collection of the volatile fraction. The OBEO collected in the Clevenger receiver was subsequently subjected to liquid–liquid extraction with petroleum ether to facilitate separation of the essential oil from residual water. The petroleum ether phase containing the essential oil was carefully separated, and the solvent was removed under reduced pressure by vacuum distillation. The recovered essential oil was then subjected to gravimetric determination of yield as given in Equation (3).

(3) $${Yield}_{OBEO}=(\frac{M_{sample}-M_{NaDES}}{Weight\;of\;blend\times \left(1-X\right)})\times 1000\;$$

Here,$\;M_{NaDES},\;M_{sample}$ and X denote weight gain in collector for NaDES (blank), weight gain in collector of *O. basilicum +* NaDES, and water level of *O. basilicum* leaves, respectively.

### 4.5. Initial Trials

One factor at a time was conducted to find the levels of parameters selected in Table 3. In this method, the effect of an individual variable was tested on the target response while keeping all other parameters at baseline (fixed levels) [21]. In the present study, the factors studied were NaDES percentage (0–70%), liquid-to-solid ratio (5.5–10) mL/g, extraction temperature (70–120) °C, and extraction time (70–120) min as described in Table 1. During the whole experiment, each factor was applied over a wider range while keeping the rest constant at predetermined values: 2.75% NaDES, 7.75 mL/g (solvent-to-solid ratio), 90 °C temperature, and 270 min. The outcomes of these initial experiments help us apply Design of Experiment (DoE) model, CCD in RSM.

### 4.6. Design of Experiment Layout

In the DoE for optimization of OBEO, four independent variables, including percentage NaDES (A), liquid-to-solid ratio (mL/g) (B), temperature °C (C), and distillation time (D) were applied at different levels as shown in Table 3. The fractionally CCD was augmented by conducting 5, 8, and 8 experiments at central, factorial, and axial points, respectively, to carry out the extraction of OBEO, and the outcomes were analyzed using Design-Expert 13 (Stat-Ease Inc., Minneapolis, MN, USA) for the analysis of variance (ANOVA) and adequacy of the applied DoE. After initial checkouts, the yield of OBEO (mL/Kg of fresh leaves) was modulated following the second-order regression equation (Equation (4)).

(4) $$y=\beta_{o}+\sum_{i=1}^{k}\beta_{i}X_{i}+\sum_{i=1}^{k}\beta_{ii}X_{i}^{2}+\Sigma_{i=1}^{k}\;\sum_{j=i+1}^{k-1}{\;\beta }_{ij}\;XiX_{j}+€$$

Here Xi *β*, and *€* represent distillation factors applied, regression coefficients and pure error, respectively.

### 4.7. Artificial Neural Networking (ANN)

**Figure 9:**
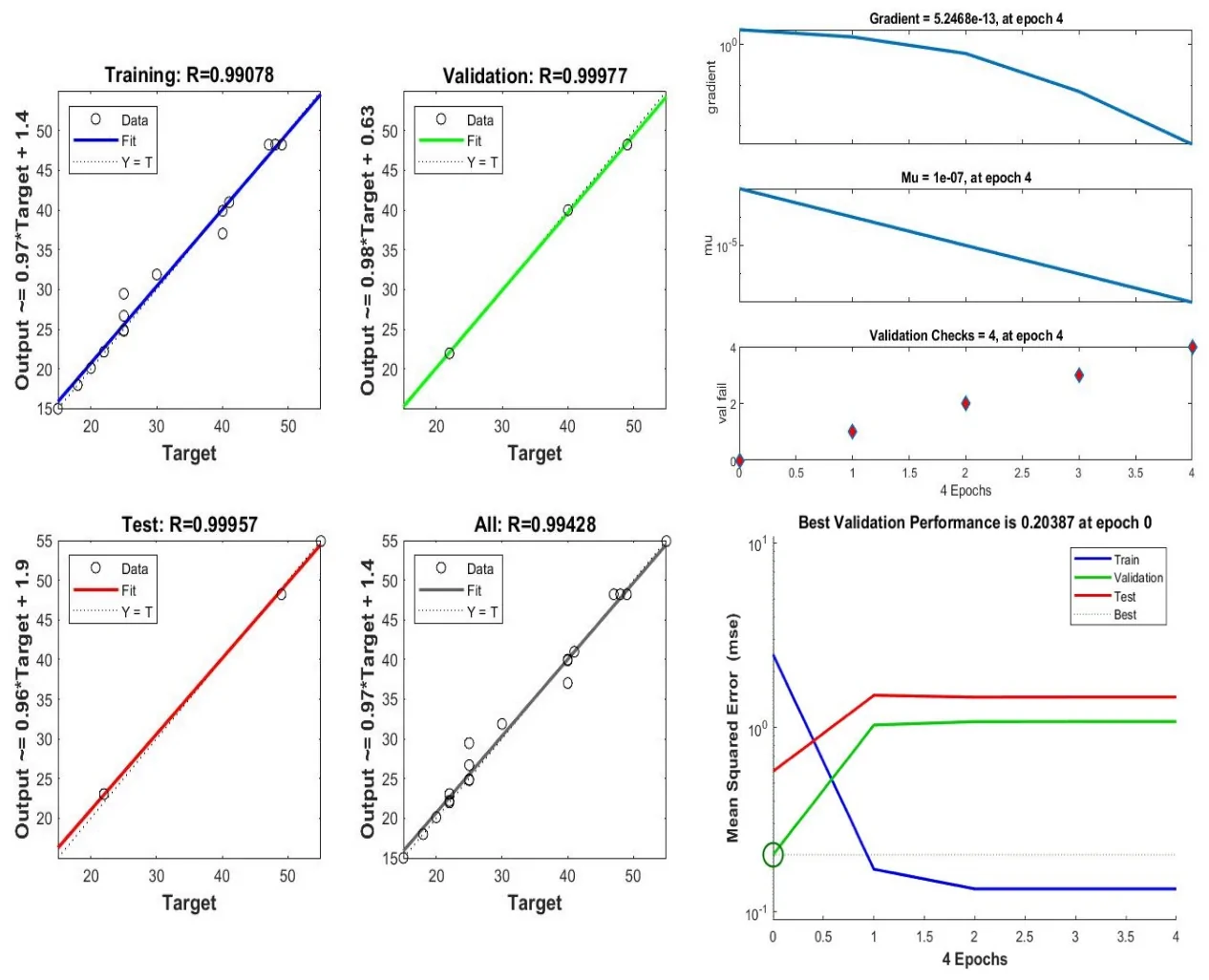
Artificial neural networking-based regression graphs for the yield of OBEO using NaDES-based distillation.

The nonlinear relationship between the process variables, including NaDES percentage, liquid-to-solid ratio, heating-mantle set-point temperature, and distillation time, and the OBEO yield was modeled using an artificial neural network (ANN). The experimental data generated from the RSM-based central composite design were used as the input dataset for ANN development. The dataset was divided into training (70%), validation (15%), and testing (15%) subsets. A feed-forward back-propagation neural network consisting of four input neurons, one hidden layer containing 20 neurons, and one output neuron was employed. The number of neurons in the hidden layer was selected through preliminary trial-and-error evaluation based on the predictive performance of the network [42]. Model training was performed using the Levenberg–Marquardt back-propagation algorithm implemented in the MATLAB Neural Network Toolbox (nntool). During training, the network weights and biases were iteratively adjusted to minimize the mean squared error between the experimentally observed and predicted OBEO yields. The training, validation, and test performances were evaluated using the regression coefficient (R) and mean squared error (MSE). The ANN architecture and the relationship between the input variables and predicted OBEO yield are presented in Figure 9.

### 4.8. GC-MS Analysis

The chemical composition of OBEO was analyzed using gas chromatography–mass spectrometry (GC-MS) by following our established method [16]. The essential oil extracted using NaDES, as well as control, was mixed separately with an equal volume of ether (dimethyl), and 2.0 μL of the organic layer from each solution was immediately injected into a gas chromatographic (GC) system equipped with a cross-bond diphenyl/dimethyl (5/95) polysiloxane-based capillary column (SH-Rxi^®^-5 Sil MS) having dimensions of 30 m length, 0.25 mm i.d. and 0.25 μm thickness. The column oven temperature was maintained at 80 °C for the first 3.0 min then gradually increased to 320 °C at a ramp rate of 15 °C/min and finally held at 320 °C for 3.0 min. The injection compartment temperature was maintained at 200.0 °C. The mode of injection was split type with a split ratio of 80:20. The flow rate of carrier gas was constant with pressure: 100.0 kPa, column flow: 1.46 mL/min and linear velocity of 44.5 cm/s. The chromatographed components were identified and quantified through a mass spectrometer (quadrupole, QP-2010 MS) detector equipped with an electron impact ionization source operated at source temperature: 200.0 °C; interface temperature: 250.0 °C, and acquisition at *m*/*z*: 42.0–550 amu.

### 4.9. Antimicrobial Activity

The agar well diffusion method reported by Balouiri, Sadiki [43], with some modifications, was used for the estimation of antimicrobial activity of OBEO under optimum conditions. Muller–Hinton agar was prepared using 2 g of nutrient broth mixed with 3 g agar in 200 mL of distilled and sterilized water. A separate nutrient broth was also prepared using 0.7 g of it in 50 mL of water. After dispensing 10 mL aliquots into test tubes, both media and glassware were sterilized in an autoclave at 121 °C for 90 min. The sterile Petri dishes were filled with molten agar, allowed to solidify, and then inoculated with Gram-positive (*S. aureus* and *B. subtilis*) and Gram-negative (*Escherichia coli*) strains. After that, wells were created and samples were poured. The OBEO concentration between 10 and 100% *v*/*v* of pure EO in ethanol was tested for antimicrobial activity in comparison to controls. Antimicrobial testing of OBEO was done using the agar well diffusion technique on the chosen bacterial samples. Ciprofloxacin (1000 ppm) was the positive control. Negative controls were distilled water, ethanol, and DMSO. There were no zones of inhibition observed from the negative controls, which proved that the antimicrobial effect was due to the extract of OBEO. Experiments were carried out in triplicate, and measurements were done in millimeters. All plates were placed in the dark for one day at 37 °C, and activity was assessed by measuring the area/diameter of inhibition zones. MIC value was calculated by following our previously reported protocol at a series of concentrations/volumes.

### 4.10. Antioxidant Activity

The antioxidant potential of OBEO obtained under optimum conditions was established against the DPPH free radicals as documented by Rasheed, Perveen [44]. Briefly, 1.0 milligrams of DPPH was added to 90 mL of methanol and further diluted until the absorbance read 0.98 at 517 nm. Different volumes between 200 and 1000 µL of OBEO were added to separate dark brown color vials and incubated with 200 µL of DPPH for 10 min in the dark. After incubation, the absorbance was measured at 517 nm to calculate the percentage of free radicals neutralized. The 200 µL of 100 ppm ascorbic acid was applied as a reference (positive control). The results were reported as OBEO concentration effective for half-maximal inhibition of DPPH free radicals (EC_50_).

### 4.11. Mosquito Repellent

Mosquito-repellent activity of OBEO obtained through an established protocol was estimated following the procedure reported by Giorgi, De Marinis [45] after certain modifications. A transparent polyethene cubic box (17.5 cm × 12.5 cm) was filled with 50 mosquitoes (identified as *Aedes aegypti*), applying black cotton swabs soaked in lactic acid and urea. To estimate the repellent activity, cotton swabs were soaked with 200, 400, 600, 800, and 1000 µL of OBEO and 200 µL of 100 ppm N,N-Diethyl-meta-toluamide as (DEET) as a standard mosquito repellent. The number of mosquitoes within 10 cm of the application area was counted to calculate percentage repellent (Equation (5)) and half-maximal repellent concentration (RC_50_).

(5) $$Mosquito\;repellent\;activity\;(\%)=\left(1-\frac{mosquitoes\;for\;OBEO-mosquitoes\;for\;Deet\;}{mosquitoes\;for\;lacticacid+urea}\right)\times 100$$

### 4.12. Statistical Analysis

The statistical evaluation of OBEO yield and its properties observed under each experimental condition assembled in Table S1 was performed using Design-Expert version 13 at 95% confidence level. Specifically, ANOVA was conducted to screen out the factors significantly (*p* ≤ 0.05) affecting the recovery of OBEO during NaDES-based distillation. Likewise, percentage absolute average deviation (% AAD), percentage prediction error (PPE), regression coefficient (R^2^), relative standard deviation (% RSD), and root mean square error (RMSE) of the result predicted by RSM and ANN were established using MATLAB 2019a. In addition, Tukey’s honestly significant difference (HSD) test was applied to compare the values of antioxidants and biological activities.

## 5. Conclusions

The present study successfully proves that glucose–citric acid NaDES, when coupled with distilled water in Clevenger type apparatus, worked as a more efficient extractant for the essential oil of *Ocimum basilicum* L. The optimization was done using RSM and ANN, in which both techniques successfully modeled the key extraction outcomes, where ANN proved to be slightly more efficient in the optimization of yield. Under the optimal conditions involving NaDES equal to 31.6%, solvent-to-solid ratio 7.18 mL/g, temperature 70 °C, and time 210 min offered the maximum essential oil yield of 41 mL/Kg of fresh *O. basilicum* leaves. The GC-MS analysis of OBEO extracted through the developed protocol in comparison to control indicates the efficiency of protocol for customizing chemical composition with enhanced recovery. The extracted oil showed broad-spectrum antimicrobial activity, potent antioxidant capability (90.36% inhibition at 1000 µL), and promising mosquito-repellent activity, with an IC_50_ of 407 µL. These findings indicate that glucose–citric acid–water-based NaDES is a sustainable and efficient alternative to conventional hazardous solvents, offering a valuable framework for industrial-scale, green extraction of bioactive enriched essential oils. There is potential use for the extracted *O. basilicum* essential oil in the pharma, food, cosmetic, and green pest control industries because of the presence of antioxidants, anti-microbial, and mosquito-repellent properties in the extracted oil. Further research should be done on the scale-up process, safety and toxicity of the oil, stability of the extracted oil, as well as more NaDES solutions, for better extraction efficiency.

## Figures and Tables

**Table 1:** ANOVA and fit statistics for the quadratic model followed for OBEO using citric acid and glucose-based DES.

Source	Sum of Squares	Df	Mean Square	F-Value	*p*-Value
Model	3128.57	14	223.47	69.69	* <0.0001
A-NaDES	2.00	1	2.00	0.6237	0.4597
B-L/S	18.00	1	18.00	5.61	0.0556
C-temp	233.28	1	233.28	72.74	0.0001
D-time	2.00	1	2.00	0.6237	0.4597
AB	20.48	1	20.48	6.39	0.0449
AC	0.0000	1	0.0000	0.0000	1.0000
AD	58.32	1	58.32	18.19	0.0053
BC	162.00	1	162.00	50.52	0.0004
BD	13.52	1	13.52	4.22	0.0858
CD	18.00	1	18.00	5.61	0.0556
A^2^	1040.78	1	1040.78	324.55	<0.0001
B^2^	1235.93	1	1235.93	385.41	<0.0001
C^2^	1.06	1	1.06	0.3294	0.5868
D^2^	111.32	1	111.32	34.71	0.0011
Residual	19.24	6	3.21		
Lack of Fit	4.44	2	2.22	0.6001	0.5917
Pure Error	14.80	4	3.70		
Cor Total	3220.67	20			
S.D.	1.44				
C.V. %	5.30				
R^2^	0.9939				
R^2^ (adj)	0.9796				
R^2^ (Pre)	0.8338				

* The *p* ≤ 0.05 was followed to screen out statistically significant terms.

**Table 2:** Bioactive compounds identified in OBEO through GC–MS analysis and their similarity index with reference available at NIST mass spectral library.

Peak #	Retention Time (min)	Area	%Area	Compound Name (Library)	Similarity Index (SI)
1	7.53	148,906	1.95	Limonene	91
2	7.58	167,573	2.19	Eucalyptol	91
3	8.74	1,858,137	24.32	Linalool	96
4	10.34	1,045,475	13.68	4-Allylanisole	96
5	13.35	293,235	3.84	Isocaryophyllene	91
6	13.46	322,311	4.22	Caryophyllene	91
7	13.54	132,685	1.74	2-Norpinene	88
8	16.09	457,820	5.99	di-exo-T-Cadinol	91
9	17.89	181,923	2.38	Isopropyl Myristate	92
10	18.92	121,758	1.59	Methyl isohexadecanoate	92
11	20.50	127,689	1.67	11,14-Eicosadienoic acid, methyl ester	89
12	20.554	429,754	5.62	Methyl cis-6-octadecenoate	92

Compound assignments are based on comparison of EI mass spectra with the NIST mass spectral library. Similarity index (SI) represents the degree of spectral matching with the corresponding NIST library entry.

**Table 3:** The actual and coded levels of factors applied for NaDES-based distillation of OBEO.

Factors	Variables	Units	Levels				
			Low (−α)	Low (−1)	Centre (0)	High (+1)	High (+α)
A	NaDES in water	%	5	12.5	27.5	42.5	50
B	Liquid-to-Solid ratio	mL/g	5.5	6.25	7.75	9.25	10
C	Extraction temperature	°C	60	70	90	110	120
D	Extraction time	Min	180	210	270	330	360

## Data Availability

The original contributions presented in this study are included in the article/Supplementary Materials. Further inquiries can be directed to the corresponding author.

## Supplementary Materials

The following supporting information can be downloaded at: https://www.mdpi.com/article/10.3390/ijms27177692/s1.
