# Supplementary material for: Hybrid Application of RSM and ANN to Optimize Natural Deep Eutectic Solvent-Based Distillation of Ocimum basilicum Essential Oil and Its Biological Activities
Source: Int J Mol Sci. 2026 Aug 27;27(17):7692. doi: 10.3390/ijms27177692 (PMC13565859; doi:10.3390/ijms27177692)
Supplement: Supplementary file 1 [file ijms-27-07692-s001.zip › ijms-4431906-supplementary.pdf]

Article

Hybrid Application of RSM and ANN to Optimize Natural Deep Eutectic Solvent-Based Distillation of *Ocimum basilicum* Essential Oil and Its Biological Activities

Iqra Khan [1], Sumia Akram [1], Zainab Liaqat [2], Faizan Waseem Butt [2], Haroon Iftikhar [2], Noor Ul Ain Khalid [2], Rizwan Ashraf [3,4], Imad A. Abu-Yousef [3,*], Amin F. Majdalawieh [3], Raed A. Al-Qawasmeh [5,6,7], Mohamed El-Naggar [5,6] and Muhammad Mushtaq [2,*]

[1] Division of Science and Technology, University of Education, Lahore-54000, Pakistan; iqrakhan8417@gmail.com (I.K.); sumia.akram@ua.edu.pk (S.A.)

[2] Department of Chemistry, Government College University, Lahore-54770, Pakistan; zainabliaqat1@gmail.com (Z.L.); ravianfaizan97@gmail.com (F.W.B.); chemistharoon@yahoo.com (H.I.); chemistnoor@yahoo.com (N.U.A.K.)

[3] Department of Biology, Chemistry and Environmental Sciences, American University of Sharjah, Sharjah P.O. Box 26666, United Arab Emirates; rashraf@aus.edu (R.A.); amajdalawieh@aus.edu (A.F.M.)

[4] Department of Chemistry, University of Agriculture, Faisalabad 38040, Pakistan

[5] Department of Chemistry, University of Sharjah, Sharjah P.O. Box 27272, United Arab Emirates; ralqawasmeh@sharjah.ac.ae (R.A.A.-Q.); melnagrr@sharjah.ac.ae (M.E.-N.)

[6] Advanced Biotechnology Center, Research Institute for Sciences and Engineering, University of Sharjah, Sharjah P.O. Box 27272, United Arab Emirates

[7] Department of Chemistry, The University of Jordan, Amman 11942, Jordan

* Correspondence: iabuyousef@aus.edu (I.A.A.-Y.); muhammad.mushtaq@gcu.edu.pk (M.M.)

Supplementary Data

Table S1. Experimental results along with RSM and ANN predicted yields of OBEO obtained while applying glucose/citric acid DES-based distillation.

Std	Run	Space Type	Independent Variables				Response		
			A: DES	B: L/S	C: Temp	D: Time	Yield Experimental	Yield RSM predicted	Yield ANN predicted
			%	mL/g	˚C	Min			
14	1	Axial	27.5	7.75	120	270	**55**	55	54
10	2	Axial	50	7.75	90	270	25	26	25
20	3	Center	27.5	7.75	90	270	48	48	48
18	4	Center	27.5	7.75	90	270	49	48	47
21	5	Center	27.5	7.75	90	270	49	48	49
11	6	Axial	27.5	5.5	90	270	25	26	25
12	7	Axial	27.5	10	90	270	18	19	18
1	8	Factorial	42.5	9.25	110	210	30	30	29
8	9	Factorial	12.5	6.25	70	210	22	21	22
9	10	Axial	5	7.75	90	270	25	26	25
17	11	Center	27.5	7.75	90	270	47	48	47
19	12	Center	27.5	7.75	90	270	49	48	49
5	13	Factorial	42.5	6.25	70	330	22	21	22
13	14	Axial	27.5	7.75	60	270	41	42	40
4	15	Factorial	12.5	9.25	70	330	22	21	22
7	16	Factorial	12.5	9.25	110	330	40	40	40
2	17	Factorial	42.5	9.25	70	210	**15**	14	15
15	18	Axial	27.5	7.75	90	180	40	41	41
3	19	Factorial	42.5	6.25	110	330	25	25	25
16	20	Axial	27.5	7.75	90	360	40	41	40
6	21	Factorial	12.5	6.25	110	210	20	20	19

Table S2. Comparison of RSM and ANN models based on experimental and predicted yield values and statistical parameters.

Parameters	Value
Experimental Yield (mL)	41.00 ± 0.27
Predicted Yield (RSM)	43.83
Predicted Yield (ANN)	39.81
% RSD (RSM / ANN)	4.72 / 2.08
PPE (RSM / ANN)	6.90 / 2.90
RMSE (RSM / ANN)	0.85 / 0.65
R^2 (RSM / ANN)	0.9954 / 0.9977
% AAD (RSM / ANN)	2.55 / 0.91

Table S3. Bioactive compounds identified in OBEO (extracted through hydro-distillation) through GC-MS analysis.

Peak #	Retention Time (min)	Area	%Area	Compound Name (Library)	Similarity Index (SI)
**1**	6.97	539116	5.08	Eucalyptol	89
**2**	7.85	4420041	41.66	Linalool	93
**3**	8.52	166936	1.57	Camphor	83
**4**	9.08	1897682	17.88	Anisole	93
**5**	11.36	993516	9.36	Caryophyllene	91
**6**	14.7	322366	3.04	Isopropyl Myristate	88
**7**	15.47	271092	2.55	methyl isoheptadecanoate	87
**8**	16.68	600997	5.66	10-Octadecenoic acid, methyl ester	89

Similarity index is a matching library score to NIST library.

Table S4: MIC determination of OBED extract (% v/v) against selected bacterial strains.

**Bacterial strain**	**100% (v/v)**	**50 % (v/v)**	**25 % (v/v)**	**12.5 % (v/v)**	**6.25 % (v/v)**	**3.125 % (v/v)**	**1.56 % (v/v)**	**MIC% (v/v)**
S. aureus	+	+	+	+	-	-	-	12.5
B. subtilis	+	+	+	+	+	-	-	6.25
P. multocida	+	+	+	+	+	+	-	3.125
E. coli	+	+	+	+	-	-	-	12.5

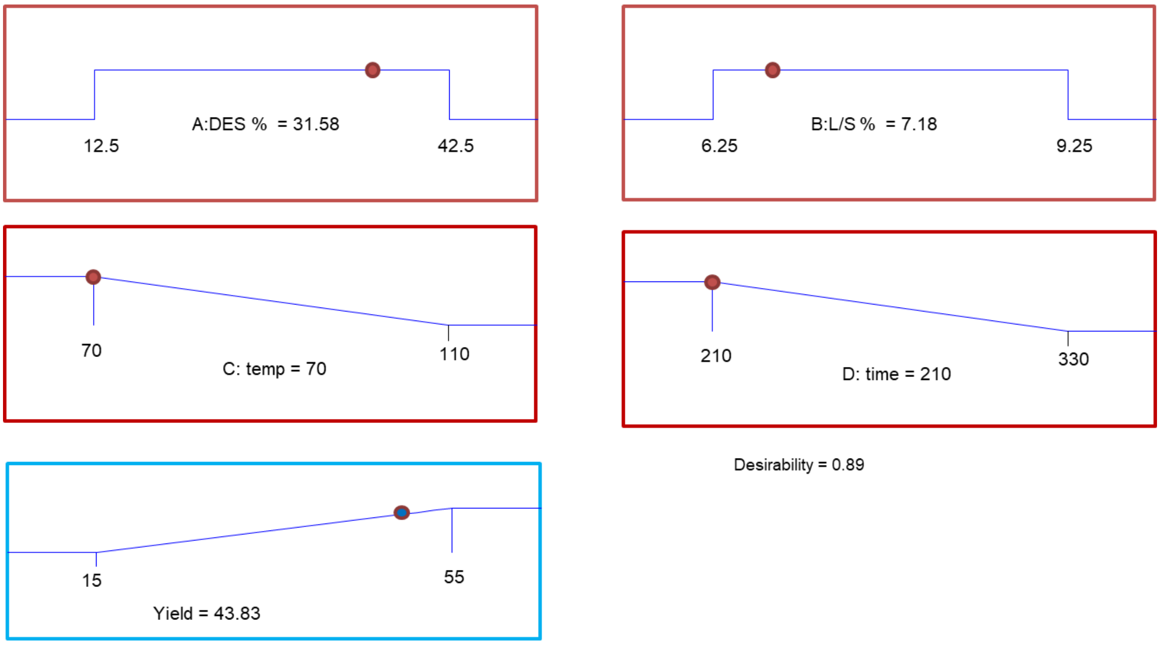

Figure S1. The desirability ramp validated for optimized recovery of OBEO applying glucose/citric acid NaDES-based distillation.

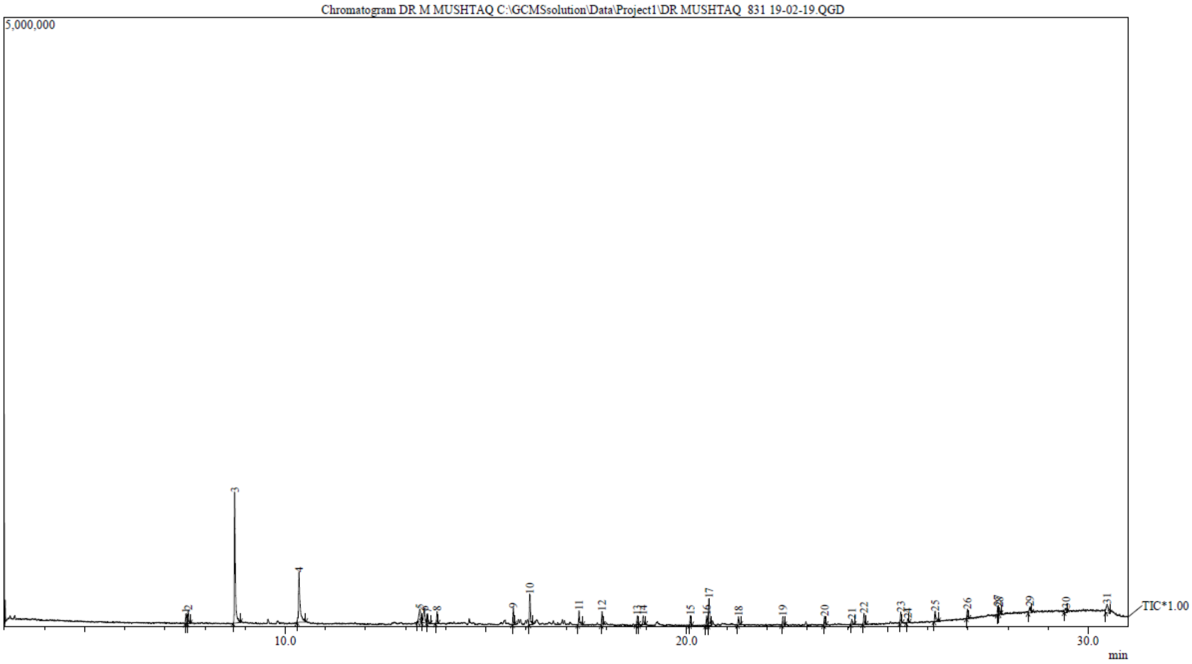

Figure S2. GC-MS chromatogram for OBEO.

Data File : C:\GCMSsolution\Data\Project1\DR.MUSHTAQ 831 19-02-19.QGD
Method File : C:\GCMSsolution\Data\Project1\ONE 2012-06-29 R 11 S 50.qgm

Library

<< Target >>
Line#:1 R.Time:7.533(Scan#:545) MassPeaks:26
RawMode:Averaged 7.525-7.542(544-546) BasePeak:68.05(11617)
BG Mode:Calc. from Peak

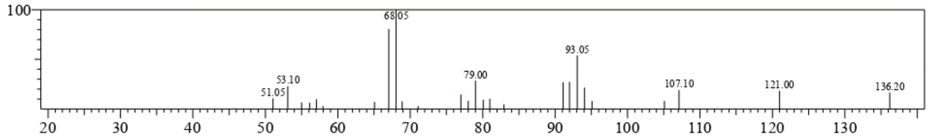

Hit#:1 Entry:6359 Library:NIST27.LIB
SI:91 Formula:C10H16 CAS:5989-54-8 MolWeight:136 RetIndex:0
CompName:Cyclohexene, 1-methyl-4-(1-methylethenyl)-, (S)-

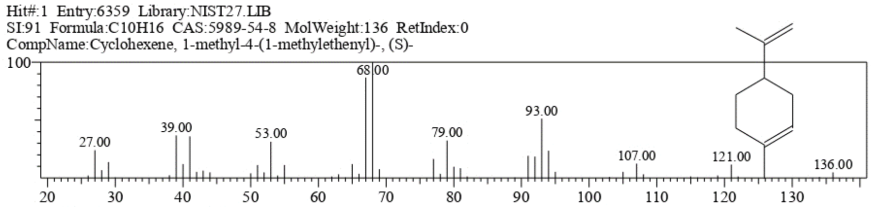

<< Target >>
Line#:2 R.Time:7.583(Scan#:551) MassPeaks:36
RawMode:Averaged 7.575-7.592(550-552) BasePeak:81.05(7131)
BG Mode:Calc. from Peak

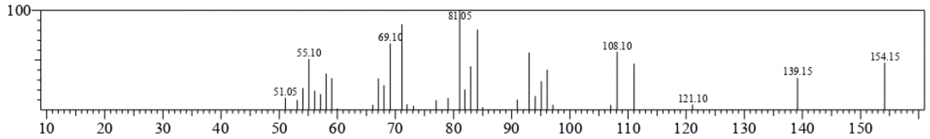

Hit#:1 Entry:9396 Library:NIST27.LIB
SI:91 Formula:C10H18O CAS:470-82-6 MolWeight:154 RetIndex:0
CompName:Eucalyptol

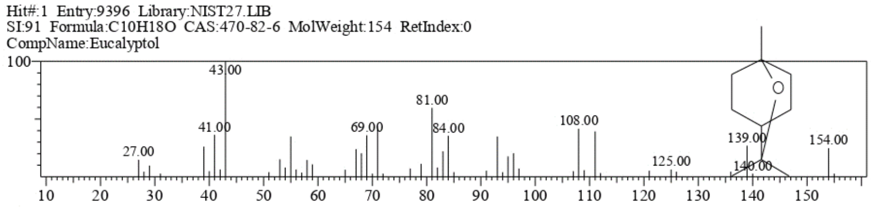

<< Target >>
Line#:3 R.Time:8.742(Scan#:690) MassPeaks:55
RawMode:Averaged 8.733-8.750(689-691) BasePeak:71.05(141798)
BG Mode:Calc. from Peak

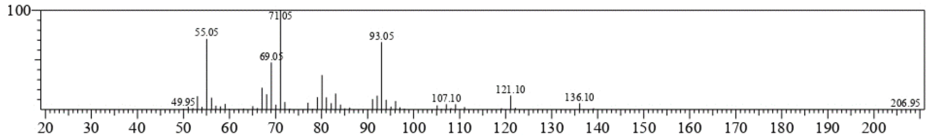

Hit#:1 Entry:9438 Library:NIST27.LIB
SI:96 Formula:C10H18O CAS:78-70-6 MolWeight:154 RetIndex:0
CompName:1,6-Octadien-3-ol, 3,7-dimethyl-

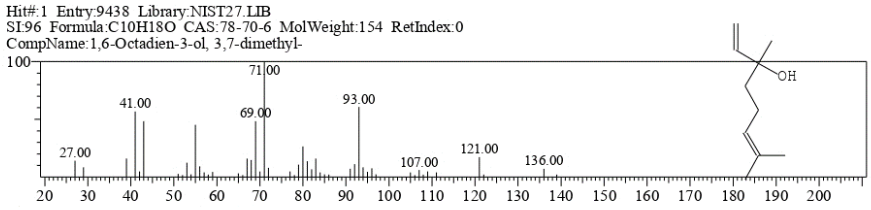

<< Target >>
Line#:4 R.Time:10.342(Scan#:882) MassPeaks:54
RawMode:Averaged 10.333-10.350(881-883) BasePeak:148.10(61340)
BG Mode:Calc. from Peak

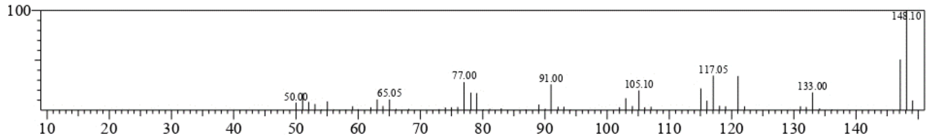

Hit#:1 Entry:8124 Library:NIST27.LIB
SI:96 Formula:C10H12O CAS:140-67-0 MolWeight:148 RetIndex:0
CompName:Anisole, p-allyl-

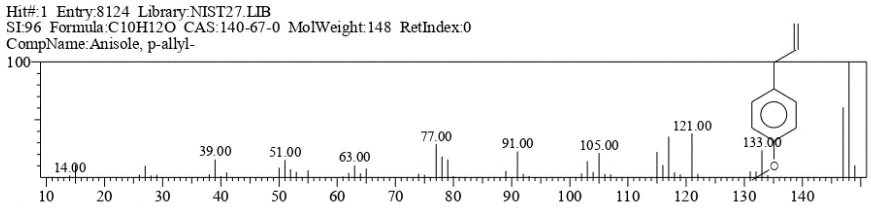

<< Target >>
Line#:5 R.Time:13.350(Scan#:1243) MassPeaks:43
RawMode:Averaged 13.342-13.358(1242-1244) BasePeak:69.05(6813)
BG Mode:Calc. from Peak

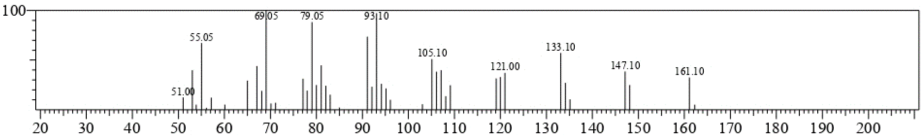

Hit#:1 Entry:16759 Library:NIST27.LIB
SI:91 Formula:C15H24 CAS:118-65-0 MolWeight:204 RetIndex:0
CompName:Bicyclo[7.2.0]undec-4-ene, 4,11,11-trimethyl-8-methylene-,[1R-(1R@,4Z,9S@)]-

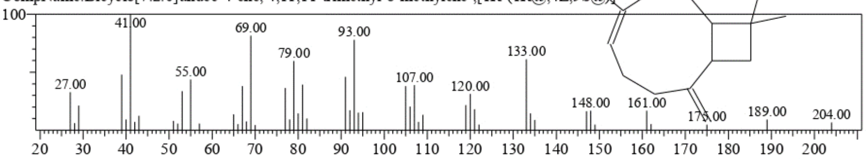

<< Target >>
Line#:6 R.Time:13.467(Scan#:1257) MassPeaks:40
RawMode:Averaged 13.458-13.475(1256-1258) BasePeak:69.15(6722)
BG Mode:Calc. from Peak

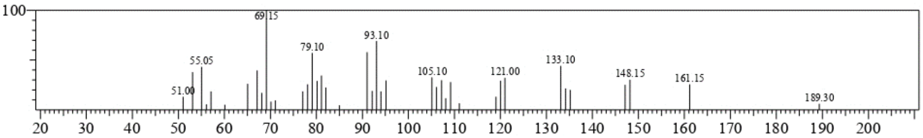

Hit#:1 Entry:16777 Library:NIST27.LIB
SI:91 Formula:C15H24 CAS:87-44-5 MolWeight:204 RetIndex:0
CompName:Caryophyllene

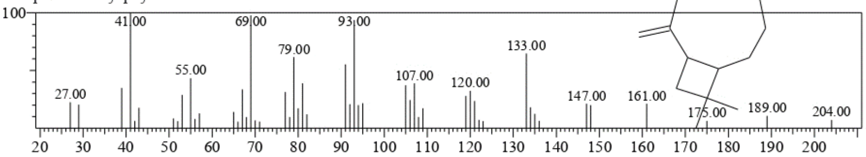

<< Target >>
Line#:7 R.Time:13.542(Scan#:1266) MassPeaks:37
RawMode:Averaged 13.533-13.550(1265-1267) BasePeak:119.05(8256)
BG Mode:Calc. from Peak

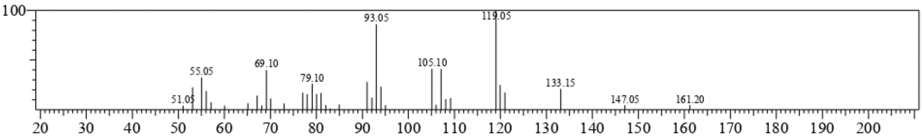

Hit#:1 Entry:42384 Library:NIST147.LIB
SI:88 Formula:C15H24 CAS:17699-5-7 MolWeight:204 RetIndex:0
CompName:Bicyclo[3.1.1]hept-2-ene, 2,6-dimethyl-6-(4-methyl-3-pentenyl)- $$ 2-Norpinene, 2,6-dimethyl-6-(4-methyl-3-pentenyl

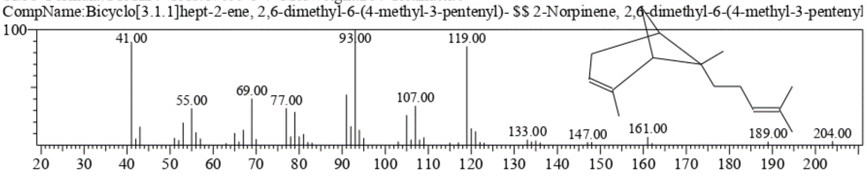

<< Target >>
Line#:10 R.Time:16.092(Scan#:1572) MassPeaks:55
RawMode:Averaged 16.083-16.100(1571-1573) BasePeak:161.15(33503)
BG Mode:Calc. from Peak

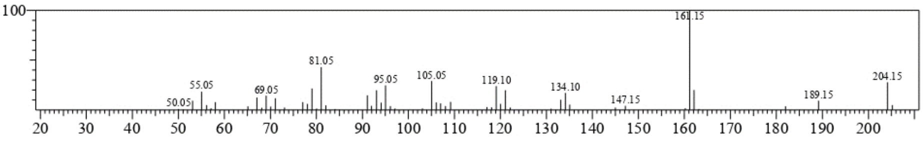

Hit#:1 Entry:52570 Library:NIST147.LIB
SI:91 Formula:C15H26O CAS:5937-11-1 MolWeight:222 RetIndex:0
CompName:.tau.-Cadinol $$ 4-Isopropyl-1,6-dimethyl-1,2,3,4,4a,7,8,8a-octahydro-1-naphthalenol # $$

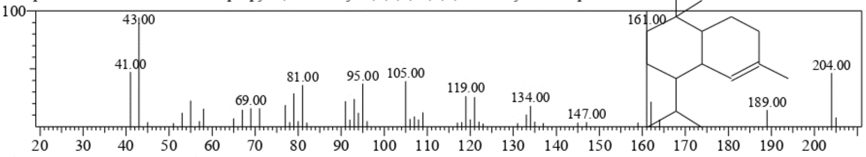

<< Target >>
Line#:12   R.Time:17.892(Scan#:1788) MassPeaks:34
RawMode:Averaged 17.883-17.900(1787-1789) BasePeak:60.00(10811)
BG Mode:Calc. from Peak

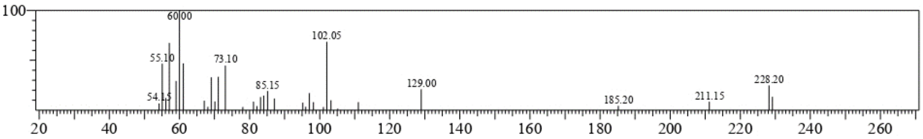

Hit#:1 Entry:22205 Library:NIST27.LIB
SI:92 Formula:C17H34O2 CAS:110-27-0 MolWeight:270 RetIndex:0
CompName:Isopropyl Myristate

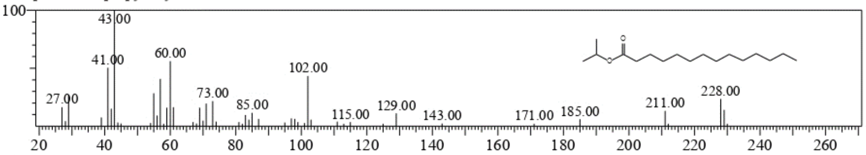

<< Target >>
Line#:14   R.Time:18.917(Scan#:1911) MassPeaks:23
RawMode:Averaged 18.908-18.925(1910-1912) BasePeak:74.10(21093)
BG Mode:Calc. from Peak

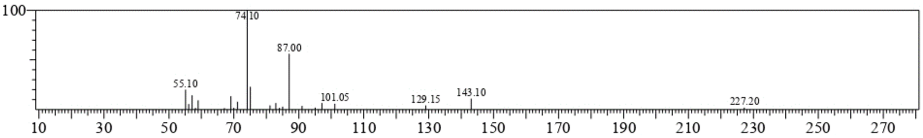

Hit#:1 Entry:22197 Library:NIST27.LIB
SI:92 Formula:C17H34O2 CAS:5129-60-2 MolWeight:270 RetIndex:0
CompName:Pentadecanoic acid, 14-methyl-, methyl ester

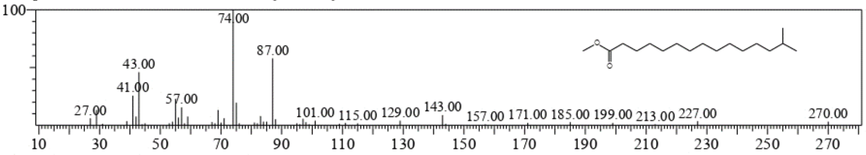

<< Target >>
Line#:16   R.Time:20.500(Scan#:2101) MassPeaks:33
RawMode:Averaged 20.492-20.508(2100-2102) BasePeak:67.05(5958)
BG Mode:Calc. from Peak

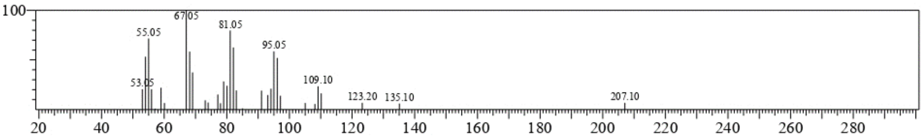

Hit#:1 Entry:104369 Library:NIST147.LIB
SI:89 Formula:C21H38O2 CAS:2463-2-7 MolWeight:322 RetIndex:0
CompName:11,14-Eicosadienoic acid, methyl ester $$ Methyl (11E,14E)-11,14-icosadienoate # $$

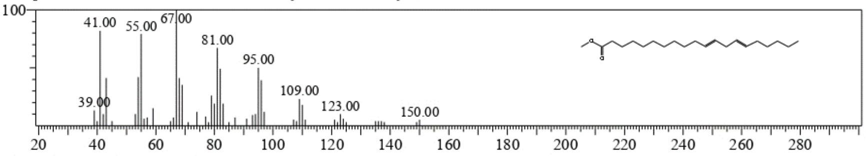

<< Target >>
Line#:17   R.Time:20.558(Scan#:2108) MassPeaks:56
RawMode:Averaged 20.550-20.567(2107-2109) BasePeak:55.10(18488)
BG Mode:Calc. from Peak

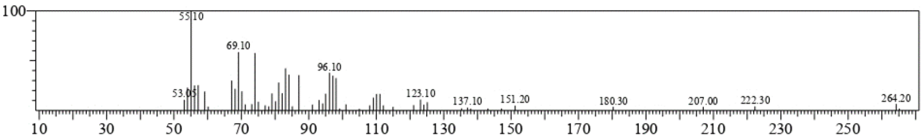

Hit#:1 Entry:23569 Library:NIST27.LIB
SI:92 Formula:C19H36O2 CAS:2777-58-4 MolWeight:296 RetIndex:0
CompName:6-Octadecenoic acid, methyl ester, (Z)-

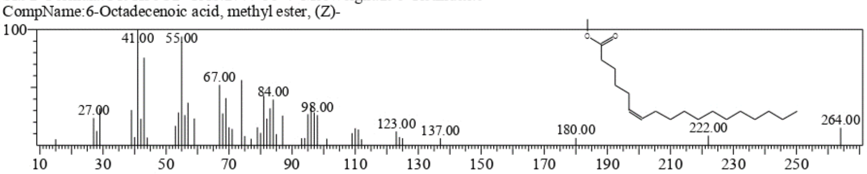

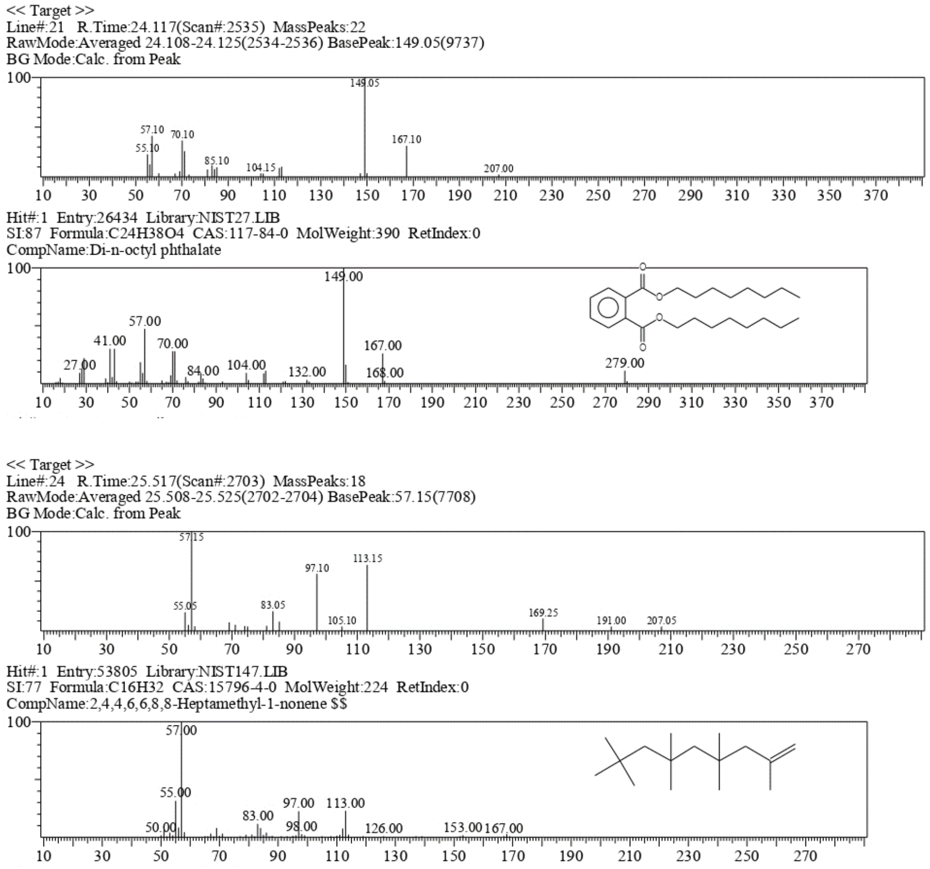

Figure S3. GC-MS library search for OBEO extracted through NaDES.

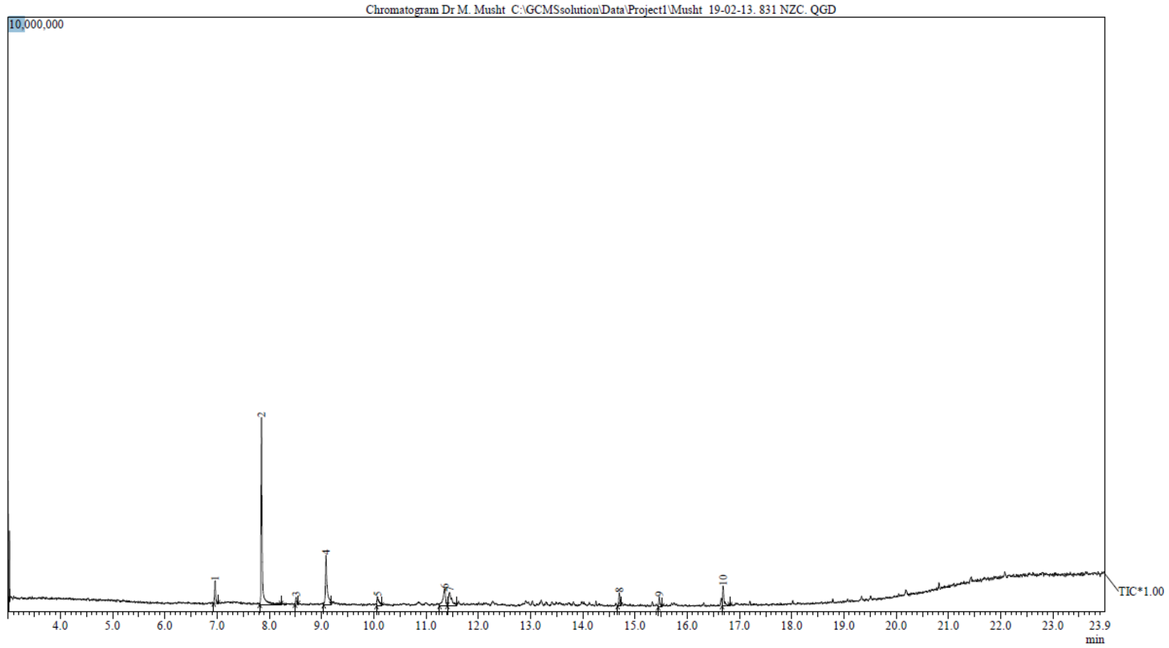

Figure S4. GC-MS chromatogram of control (OBEO extracted through hydro-distillation).

<< Target >>
Line#:1 R.Time:6.967(Scan#:477) MassPeaks:59
RawMode:Averaged 6.958-6.975(476-478) BasePeak:43.10(58226)
BG Mode:Calc. from Peak

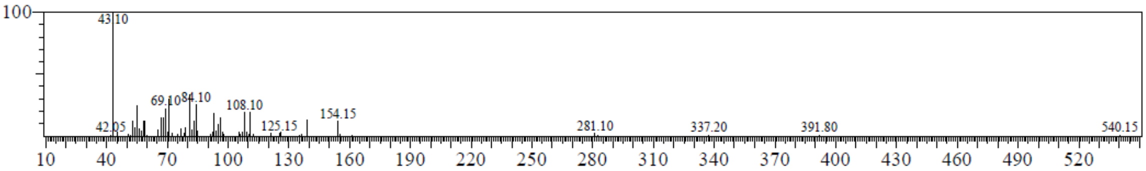

Hit#:1 Entry:9396 Library:NIST27.LIB
SI:89 Formula:C10H18O CAS:470-82-6 MolWeight:154 RetIndex:0
CompName:Eucalyptol

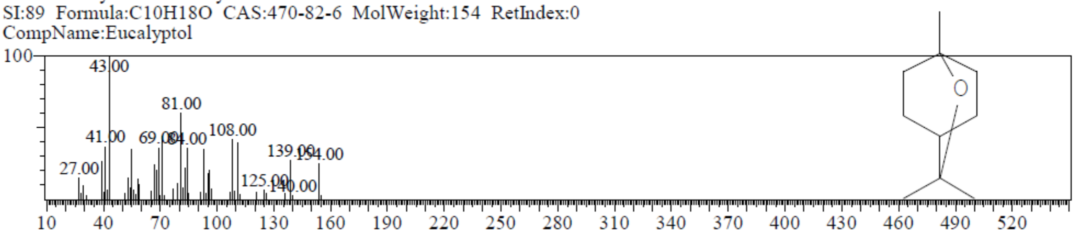

<< Target >>
Line#:2 R.Time:7.850(Scan#:583) MassPeaks:78
RawMode:Averaged 7.842-7.858(582-584) BasePeak:43.05(304110)
BG Mode:Calc. from Peak

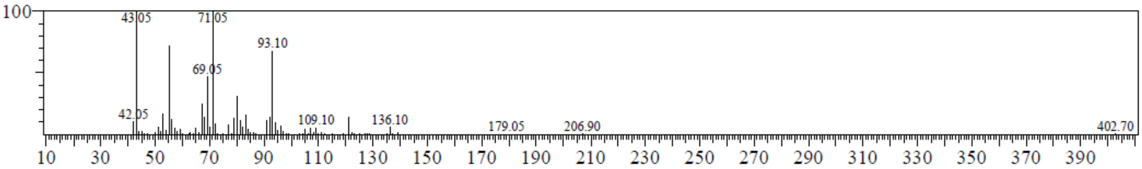

Hit#:1 Entry:9438 Library:NIST27.LIB
SI:93 Formula:C10H18O CAS:78-70-6 MolWeight:154 RetIndex:0
CompName:1,6-Octadien-3-ol, 3,7-dimethyl-

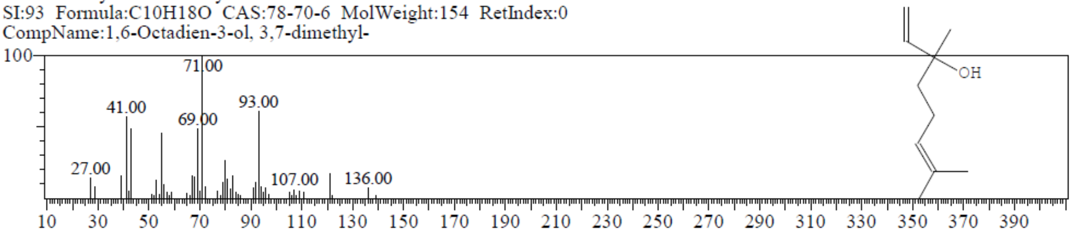

<< Target >>
Line#:3 R.Time:8.517(Scan#:663) MassPeaks:58
RawMode:Averaged 8.508-8.525(662-664) BasePeak:95.20(10341)
BG Mode:Calc. from Peak

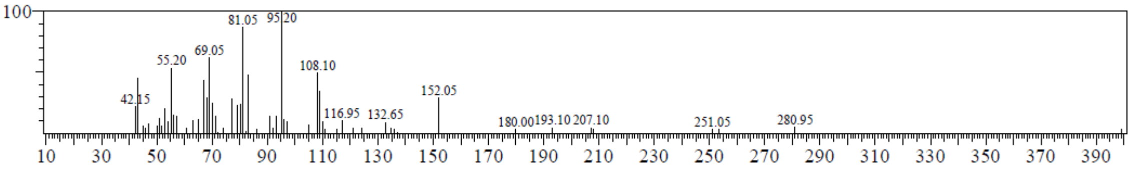

Hit#:1 Entry:9057 Library:NIST27.LIB
SI:84 Formula:C10H16O CAS:76-22-2 MolWeight:152 RetIndex:0
CompName:Camphor

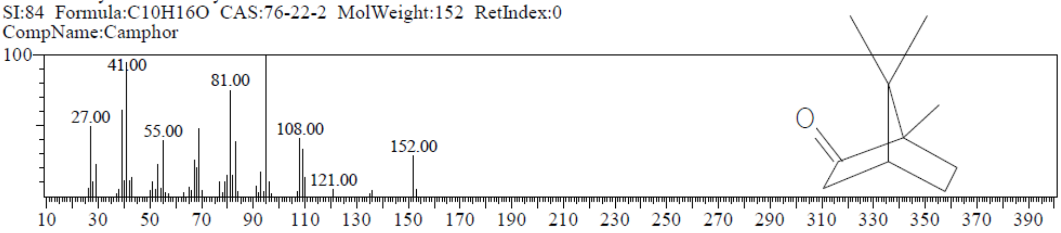

<< Target >>
Line#:4 R.Time:9.083(Scan#:731) MassPeaks:81
RawMode:Averaged 9.075-9.092(730-732) BasePeak:148.10(70614)
BG Mode:Calc. from Peak

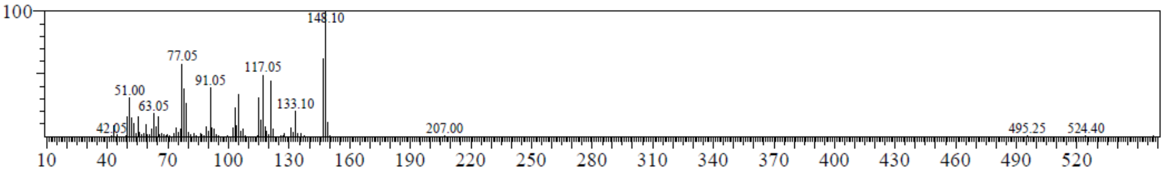

Hit#:1 Entry:14003 Library:NIST147.LIB
SI:93 Formula:C10H12O CAS:140-67-0 MolWeight:148 RetIndex:0
CompName:Anisole, p-allyl- $$ p-Allylanisole $$ p-Methoxyallylbenzene $$ Chavicol methyl ether $$ Chavicol, O-methyl- $$ Esd

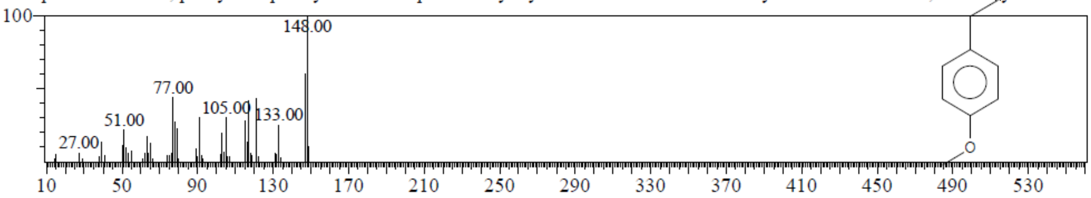

<< Target >>
Line#:6 R.Time:11.358(Scan#:1004) MassPeaks:75
RawMode:Averaged 11.350-11.367(1003-1005) BasePeak:69.05(18385)
BG Mode:Calc. from Peak

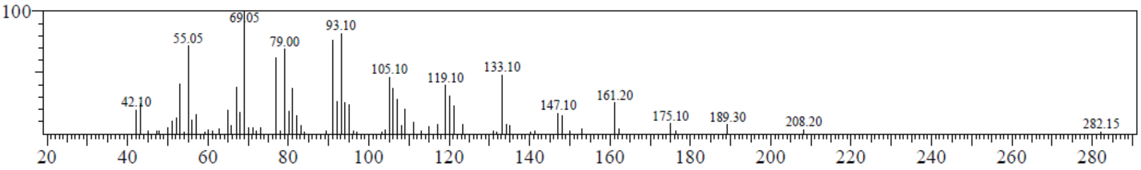

Hit#:1 Entry:16777 Library:NIST27.LIB
SI:91 Formula:C15H24 CAS:87-44-5 MolWeight:204 RetIndex:0
CompName:Caryophyllene

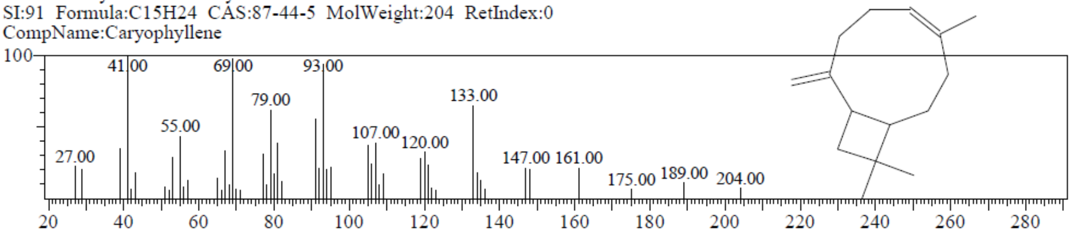

<< Target >>
Line#:8 R.Time:14.700(Scan#:1405) MassPeaks:67
RawMode:Averaged 14.692-14.708(1404-1406) BasePeak:43.10(29404)
BG Mode:Calc. from Peak

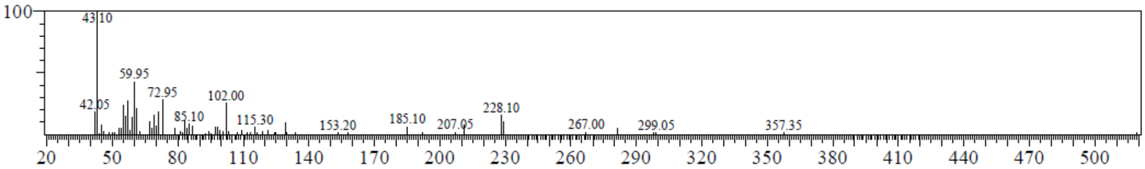

Hit#:1 Entry:22205 Library:NIST27.LIB
SI:88 Formula:C17H34O2 CAS:110-27-0 MolWeight:270 RetIndex:0
CompName:Isopropyl Myristate

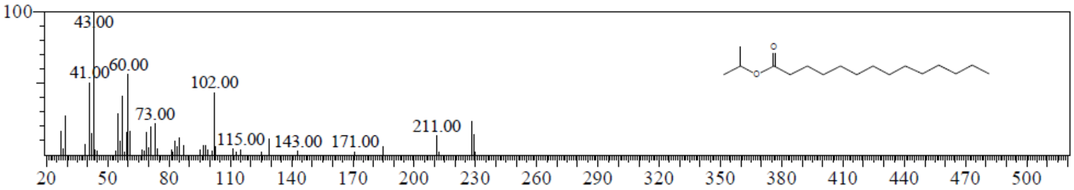

<< Target >>
Line#:9 R.Time:15.467(Scan#:1497) MassPeaks:58
RawMode:Averaged 15.458-15.475(1496-1498) BasePeak:74.10(24558)
BG Mode:Calc. from Peak

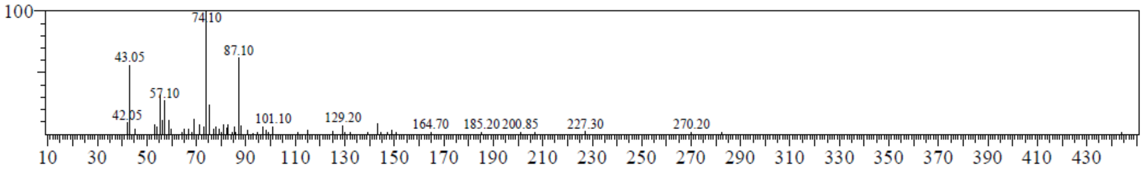

Hit#:1 Entry:22972 Library:NIST27.LIB
SI:87 Formula:C18H36O2 CAS:6929-4-0 MolWeight:284 RetIndex:0
CompName:Hexadecanoic acid, 15-methyl-, methyl ester

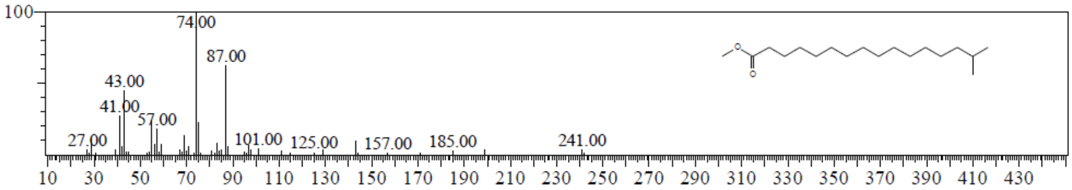

<< Target >>
Line#:10 R.Time:16.683(Scan#:1643) MassPeaks:86
RawMode:Averaged 16.675-16.692(1642-1644) BasePeak:55.10(22025)
BG Mode:Calc. from Peak

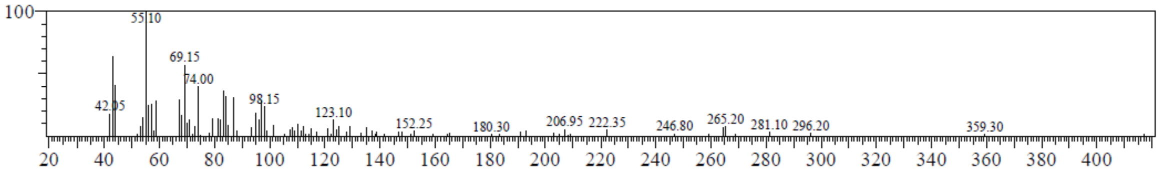

Hit#:1 Entry:91954 Library:NIST147.LIB
SI:89 Formula:C19H36O2 CAS:13481-95-3 MolWeight:296 RetIndex:0
CompName:10-Octadecenoic acid, methyl ester $$ Methyl (10E)-10-octadecenoate # $$

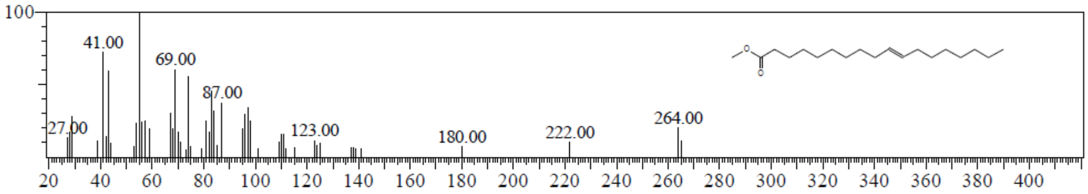

Figure S5. Library search of OBEO control (OBEO extracted through hydro-distillation).
